# Multi-model genome-wide association studies of leaf anatomical traits and vein architecture in rice

**DOI:** 10.3389/fpls.2023.1107718

**Published:** 2023-04-12

**Authors:** Supatthra Narawatthana, Yotwarit Phansenee, Bang-On Thammasamisorn, Phanchita Vejchasarn

**Affiliations:** ^1^ Rice Department, Thailand Rice Science Institute, Ministry of Agriculture and Cooperatives (MOAC), Suphan Buri, Thailand; ^2^ Ubon Ratchathani Rice Research Center, Rice Department, Ministry of Agriculture and Cooperatives (MOAC), Ubon Ratchathani, Thailand

**Keywords:** rice, leaf thickness, vein size, vein density, GWAS

## Abstract

**Introduction:**

The anatomy of rice leaves is closely related to photosynthesis and grain yield. Therefore, exploring insight into the quantitative trait loci (QTLs) and alleles related to rice flag leaf anatomical and vein traits is vital for rice improvement.

**Methods:**

Here, we aimed to explore the genetic architecture of eight flag leaf traits using one single-locus model; mixed-linear model (MLM), and two multi-locus models; fixed and random model circulating probability unification (FarmCPU) and Bayesian information and linkage disequilibrium iteratively nested keyway (BLINK). We performed multi-model GWAS using 329 rice accessions of RDP1 with 700K single-nucleotide polymorphisms (SNPs) markers.

**Results:**

The phenotypic correlation results indicated that rice flag leaf thickness was strongly correlated with leaf mesophyll cells layer (ML) and thickness of both major and minor veins. All three models were able to identify several significant loci associated with the traits. MLM identified three non-synonymous SNPs near *NARROW LEAF 1 (NAL1)* in association with ML and the distance between minor veins (IVD) traits.

**Discussion:**

Several numbers of significant SNPs associated with known gene function in leaf development and yield traits were detected by multi-model GWAS performed in this study. Our findings indicate that flag leaf traits could be improved via molecular breeding and can be one of the targets in high-yield rice development.

## Introduction

Genome-wide association studies (GWAS) have been developed into a powerful tool in underpinning the genetic architecture of complex and agriculturally important traits in many major crop species, including rice (*Oryza sativa* L.). Leaf shape, size, and thickness determine the leaf’s photosynthetic capability and have a great impact on yield, disease resistance, and stress responses in crops (Tsukaya, 2005; Pérez-Pérez et al., 2010; Wang et al., 2011; Liu et al., 2014; Fan et al., 2015). Therefore, improving leaf traits is a very important target in rice breeding.

Plants establish their leaf function *via* the precise spatial specification of specialized cell types. In rice, the outer leaf comprises epidermal pavement cells, stomatal pores, bulliform cells located at the adaxial epidermis, and trichomes, whereas the inner leaf consists of photosynthetic mesophyll cells and a network of vasculature enclosed by bundle sheaths of parenchyma cells. Rice leaf originates as lateral outgrowths from the leaf primordial cells in the shoot apical meristem (SAM), requiring a synchronized developmental process. Leaf shape is determined through cell division and expansion changes during the leaf proximodistal axis formation, tissue differentiation, and specification (Itoh et al., 2005).

The rice flag leaf has been indicated as one of the top two leaves of a rice plant that contributes over 80% of the total assimilates of rice grains (Tomoshiro et al., 1983; Gladun and Karpov, 1993; Fujii and Saka, 2001; San-oh et al., 2004). In modern rice breeding, a narrow and short leaf rather than a wide and long leaf is preferable considering the efficiency in light perception, as a short and narrow leaf has been indicated to associate more with the small leaf angle thus erect leaf type (Vangahun, 2012). The erect flag leaf can enhance light capture, boosting photosynthetic activity and yield (Yoshida, 1972; Sinclair and Sheehy, 1999). However, longer and lower stomatal density leaves tend to be more desirable and perform better under drought conditions (Kumar et al., 2021).

Leaf thickness is one of the anatomical traits that has been considered a key index in high-yield potential rice breeding (Yoshida, 1972; Peng et al., 1994; Peng, 2000). Thick leaves usually have high chlorophyll and Rubisco content together with high mesophyll conductance affecting CO_2_ diffusion in the leaves (Evans and Loreto, 2000; Terashima et al., 2001; Murchie et al., 2002; Li et al., 2011; Narawatthana, 2013). Consequently, flag leaf thickness influences the net photosynthetic rate and is positively correlated with rice grain yield (Cook and Evans, 1983; Peng, 2000; Wu et al., 2009; Takai et al., 2010; Liu et al., 2014). Compared with leaf length and width, rice leaf thickness was scarcely studied since direct measurement of leaf thickness is extremely challenging and laborious. Some indirect measurements; e.g., leaf dry matter percentage, specific leaf weight (mass per area), and specific leaf area (area per mass) had been commonly applied as a proxy in determining the leaf thickness in rice (Kanbe et al., 2008; Takai et al., 2010; Liu et al., 2014; Hoang et al., 2019).

Recently, leaf venation architecture in rice has been focused on as one of the important leaf anatomical characteristics influencing leaf gas exchange and hydraulic conductance. Vein density and size were positively correlated with leaf hydraulic conductance thus improving leaf CO_2_ assimilation efficiency which is vital for improving photosynthesis (Tabassum et al., 2016; Pitaloka et al., 2021; Ye et al., 2021). Rice leaf veins are different in size and functions and, apart from the midrib, are divided into two categories (major and minor veins) based on their size. Leaf venation architecture: i.e., vein arrangement, density, size, and geometry of xylem and phloem vessels, has enormous variation and contributes to different leaf performances (Sack and Scoffoni, 2013). Although leaf venation and leaf thickness can greatly affect photosynthetic efficiency, these traits have not been set as a major target in rice breeding programs due to lacking their genetic background.

Genes and quantitative trait loci (QTLs) related to leaf morphogenesis in rice regulate leaf shape through cell division and differentiation as well as the signaling pathways of phytohormone and transcription factors. Many genes and QTLs involved in the developmental processes of leaf morphogenesis have been identified and characterized by mutant analysis. The rice dwarf mutant named *narrow leaf 1* (*nal1*) exhibits reduced polar auxin transport capacity leading to decreased leaf width and a defective vascular system (Qi et al., 2008). Consequently, *qFW4-2*, a QTL for flag leaf width identified in a chromosomal segment substitution line (CSSL) population, was also mapped to the location corresponding to *NAL1* (Tang et al., 2018). Tang et al. (2018) confirmed the function of the gene *GRAIN NUMBER, PLANT HEIGHT, AND HEADING DATE 7.1* (*GHD7.1*) in increasing flag leaf size and photosynthetic capacity thus improving yield potential. Furthermore, a genome-wide association study (GWAS) conducted using a panel of 529 rice accessions indicated that *GHD7* and *NAL* were the major loci controlling the natural variation of chlorophyll content in rice leaves (Wang et al., 2015). Recently, GWAS was carried out for 29 rice leaf traits related to leaf size, shape, and color and unraveled several QTLs (Yang et al., 2015).

To date, gene mapping and GWAS can provide the most comprehensive investigation for identifying genes in plants for almost all traits which are complex and regulated by many genes and influenced by the environment. However, several false positives could be generated from GWAS due to population structure and family relatedness (Kaler et al., 2020). In the mixed linear models (MLM), controlling the false positives is often performed by incorporating population structure and a kinship matrix as covariates into this type of single locus model (Price et al., 2006). Nonetheless, false negatives can also be introduced by overfitting the model that might exclude some important loci. Recently, multi-locus GWAS analysis methods such as fixed and random model circulating probability unification (FarmCPU) and Bayesian-information and linkage-disequilibrium iteratively nested keyway (BLINK) have been developed to overcome the false-negative problem (Zhang et al., 2019). In this study, three different statistical models both single locus and multi-locus models: MLM, BLINK, and FarmCPU, were compared for GWAS of rice leaf thickness and venation architecture, in a genetically diverse worldwide collection called Rice Diversity Panel 1 (RDP1). The multi-model GWAS was performed to detect an association between SNP and the flag leaf traits.

## Materials and methods

### Plant material and genotypic data

The Rice Diversity Panel 1 (Reg. No. MP-6, NSL500357 MAP) (RDP1) consists of 421 rice germplasm including both Asian landraces and worldwide elite cultivars obtained from 79 countries representing the major rice growing regions (Eizenga et al., 2014). In this study, we used 329 accessions of the RDP1 containing 141 accessions belonging to the Indica varietal group including the indica (78 accessions), aus (52 accessions), and admixture of Indica (11 accessions) and 188 accessions comprising the Japonica varietal group including the tropical japonica (64 accessions), temperate japonica (87 accessions), an admixture of Japonica (24 accessions), and aromatic (13 accessions) were analyzed. **Supplemental Table S1** contains information about the accession name, accession number, original providing country, and sub-population group based on 36 SSRs and 700,000 SNPs (Ali et al., 2011; McCouch et al., 2016).

### Leaf anatomical traits and vein architecture measurements

**Figure 1:**
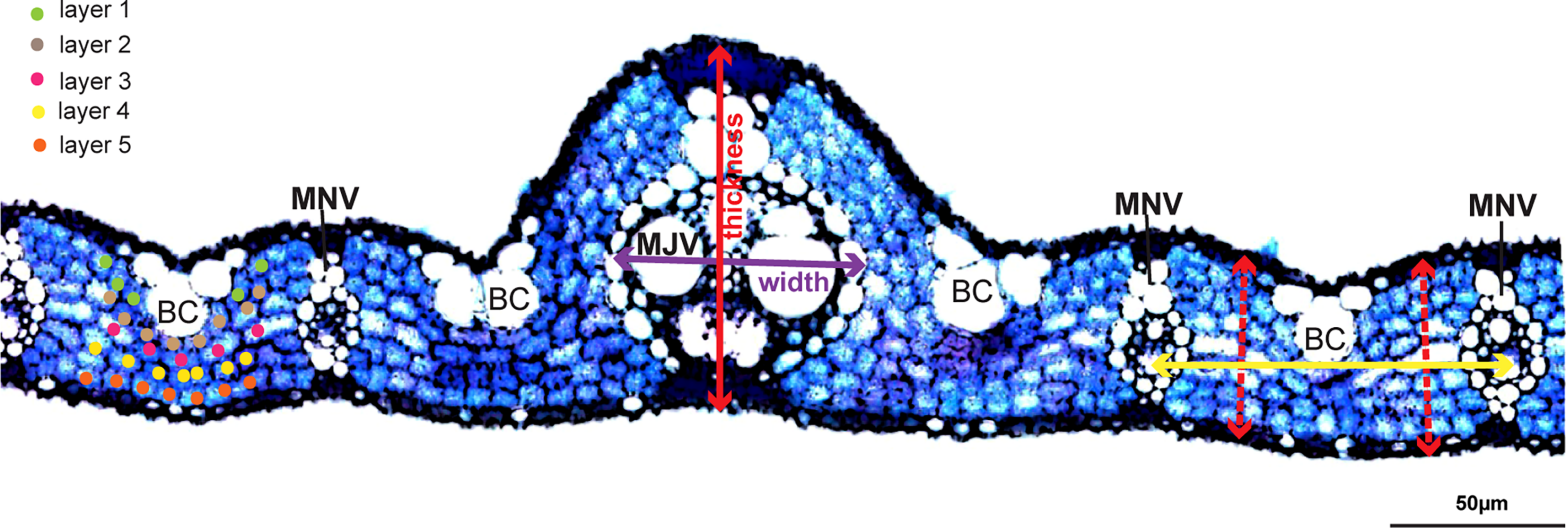
Measurement of rice leaf anatomical and vein traits. Leaf thickness, minor vein (MNV), and major vein (MJV) thickness were measured by drawing a straight line from the top of the upper epidermis to the bottom of the lower epidermis as illustrated using the red lines. Width at minor and major veins was measured along the axis as indicated with the purple line. Different color dots represent different mesophyll cell layers in the counting of mesophyll cell layer numbers. The yellow line shows the inter-veinal distance measurement between 2 adjacent minor veins. BC, Bulliform cells. Scale bar = 50 µm.

A total of 329 rice accessions of RDP1 were grown in irrigated paddy fields at Thailand Rice Science Institute, Suphan Buri, Thailand (14° 28’ 16.79” N and 100° 07’ 3.60” E) in the wet season from 2017 to 2018. Individual rice seedlings at 20 days old were manually transplanted in 3 rows per accession at a spacing of 25 cm between rows and 25 cm between plants. The N:P: K fertilizer was applied at the rate of 37.5:37.5:37.5 kg ha^-1^ before transplanting and at the rate of 37.5:0:0 kg ha^-1^ during the heading stage. Fully expanded flag leaves were collected, and the widest part of the individual leaf was excised in 1 cm long for free-hand sectioning. Leaf sections were cleared with an 85% (w/v) lactic acid in saturated chloral hydrate at 70°C for 1 hour before staining with 0.01% (w/v) toluidine blue in 15% boric acid for 10 seconds. Stained leaf sections were examined under a microscope (IX71, Olympus, Japan) and documented using a digital camera DP73 (Olympus, Japan) for image analysis using Figi (Schindelin et al., 2012). Leaf thickness (LT), major vein thickness (MJVT), minor vein thickness (MNVT), major vein width (MJVW), minor vein width (MNVW), and the number of mesophyll cell layer (ML) were measured as illustrated in **Figure 1**.

Leaf width and length were measured *in vivo*, and the stained leaf sections were used for major veins and minor veins counting. Vein density or vein length per leaf area (VLA) was calculated as follows (Ye et al., 2021).


$$\text{VLAmajor }=\;\frac{\text{number of leaf major veins }\times \text{leaf length}}{\text{leaf width }\times \text{leaf length}}$$

$$\text{VLAminor }=\frac{\text{number of leaf minor veins }\times \text{leaf length}}{\text{leaf width }\times \text{leaf length}}$$

VLA =VLAmajor+VLAminor

### Statistical analysis

Phenotypic data were analyzed using R software version 4.1.3 to estimate the means, standard deviations (sd), coefficients of variation (CV), variances, and broad-sense heritability for each trait. The broad-sense heritability (H^2^) was estimated by using the genotypic and phenotypic variances as follows: H^2^ = (F-1)/F, where F is the F-value from ANOVA for the genotype factor. Phenotypic correlations among the traits were computed by Pearson’s method, using the ‘psych’ package in R (Revelle and Condon, 2019). Multiple pairwise comparisons were performed using the Kruskal-Wallis test to examine the significant difference among sub-population groups. A pairwise Wilcoxon signed-rank test with Bonferroni correction was conducted to detect a significant difference in between two sub-population groups. All statistical graphs were created using R version 4.1.3 (R Core Team, 2022).

### Genome-wide association analysis

The association analysis was conducted using MLM (Yu et al., 2006), BLINK (Huang M. et al., 2019), and FarmCPU (Liu et al., 2016) model using the R software package Genome Association and Prediction Integrated Tool (GAPIT) version 3 (Lipka et al., 2012). The mean of each trait collected over two years was used as input data for GWAS. MLM analysis was applied using the P3D (population parameters previously determined) method without compression. A total of 700,000 SNPs were used for GWAS. The genome-wide significant *P* value threshold was adjusted based on Bonferroni correction. The SNP-trait associations were declared significant when the *P* value<1e-07 and the corrected *P* value< 0.05. Gene annotation was performed on 100 kb of the flanking regions of the associated loci. The Manhattan plots and Q-Q plots were generated using the GAPIT package in R.

### Identification of candidate genes

To explore candidate genes underlying leaf anatomical traits and vein architecture identified in this study, the SNPs with p< 0.0001 were considered as genomic regions carrying candidate genes. All gene loci within 100kb of each SNP were extracted from the annotation of the *Oryza sativa* reference sequence (OsNipponbare-Reference-IRGSP-1.03; http://rapdb.dna.affrc.go.jp/download/irgsp1.html) using the Bioconductor packages in R (Huber et al., 2015). Gene Ontology (GO) terms were retrieved from the BMRF tool (https://www.ab.wur.nl/bmrf). To validate the GWAS results, all annotated gene loci included in the genomic regions were compared with genes known to be related to the phenotypic traits analyzed available in the Oryzabase database (https://shigen.nig.ac.jp/rice/oryzabase/) or QTLs/genes present in literature. LDBlockshow (Dong et al., 2021) was used to estimate the local linkage disequilibrium (LD) block on 100 kb of the genomic region containing significant SNPs. The haplotype analysis was conducted on selected candidate genes using all SNPs within the gene coding region using Haploview 4.1 software (Barrett et al., 2005). The haplotype contains at least 10 accessions was considered a major haplotype. Kruskal-Wallis test and pairwise Wilcoxon signed-rank test with Bonferroni correction were performed to examine the significant difference in phenotypic variation among haplotypes.

## Results

### Phenotypic variation and heritability of the leaf anatomical traits and vein architecture

We measured 8 leaf and vein traits in the 329 accessions of RDP1 over two years. These traits can be categorized into 2 major groups: leaf anatomical traits (leaf thickness, number of mesophyll cell layer, and inter-veinal distance), and vein traits (major vein thickness, minor vein thickness, major vein width, minor vein width, and vein density). All the traits exhibited high values of broad-sense heritability (H^2^), ranging from 0.743 to 0.996 (**Table 1**). Heritability for mesophyll cell layer and vein density was very high in the RDP1 sub-population (0.994 and 0.996). The results revealed the extensive variation in leaf anatomical and vein traits under strong genetic control among the rice accessions. The coefficient of variance (CV) of these traits varied from 9.7% to 51.53% and VLA showed the strongest variation.

**Table 1:** Phenotypic variation and heritability on leaf anatomy and vein traits of RDP1.

Trait	Range	mean	SD	CV	H^2^
Leaf Thickness (µm)	35.72 – 91.60	55.92	9.58	17.14	0.919
Mesophyll cell layer (layers)	4.65 – 10.00	6.59	0.89	13.58	0.994
Major Vein Thickness (µm)	65.21 – 168.32	107.04	19.07	17.81	0.898
Major Vein Width (µm)	53.49 – 107.14	75.19	7.52	10.00	0.948
Minor Vein Thickness (µm)	29.60 – 85.47	46.16	9.27	20.07	0.743
Minor Vein Width (µm)	19.05 – 42.33	26.52	3.77	14.21	0.912
Inter-Veinal Distance (µm)	86.31 – 136.45	110.63	10.74	9.70	0.906
Vein length per leaf area (mm/mm^2^)	2.02 – 16.95	6.19	2.19	51.53	0.996

SD, standard deviation; CV, coefficient of variance; H^2^, heritability.

**Figure 2:**
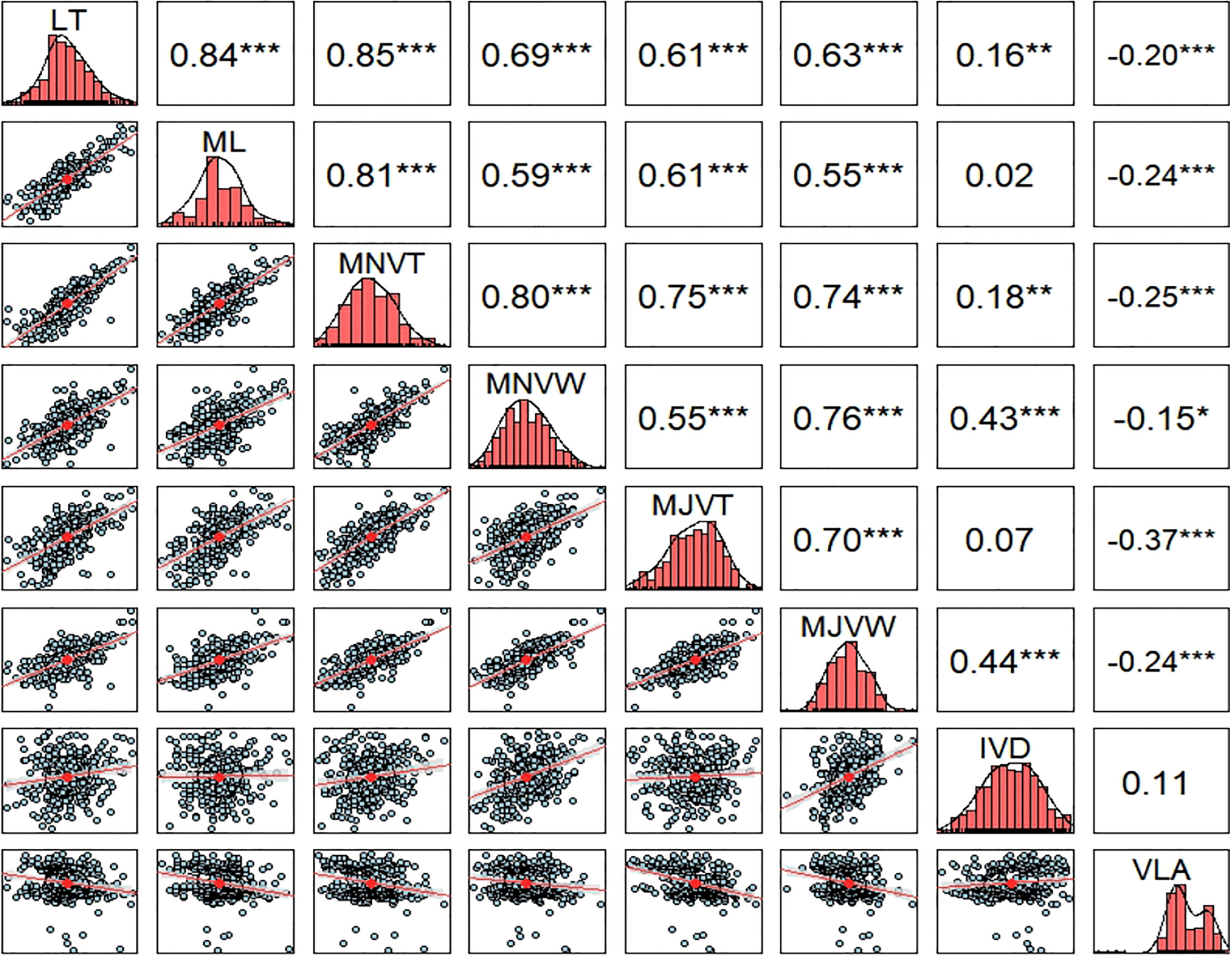
Pearson correlation coefficients between leaf and vein traits across all the rice accessions of the RDP1 subpopulation. The diagonal represents the density histogram of the trait. Asterisks indicate a significant level of Pearson correlations (*< 0.01, **< 0.005, ***< 0.001), n = 329 varieties. LT, leaf thickness; ML, number of mesophyll layers; MJVT, major vein thickness; MJVW, major vein width; MNVT, minor vein thickness; MNVW, minor vein width; IVD, inter-veinal distance; VLA, vein length per leaf area.

The phenotypic correlations among the leaf anatomical and vein traits were analyzed (**Figure 2**). LT, ML, and MNVT were strongly positively correlated, with correlation coefficients over 0.85 (*P*< 0.001), among themselves. These results indicate that rice leaf thickness is enormously influenced by the number of mesophyll cell layers and minor vein size. Here, we demonstrate a very strong positive correlation in thickness and width among major and minor veins of the RDP1 sub-population. MJVT, MJVW, and MNVW showed a relatively strong positive relationship with LT and ML, albeit less correlated than observed for MNVT. The previous report has found that thick rice leaves are supported by wider minor veins and thin rice leaves are supported by narrow minor veins (Chatterjee et al., 2016). Contrary to the previous report (Chatterjee et al., 2016), we found no significant relationship between IVD and VLA in this population. However, VLA was negatively correlated with MJVW and MNVW, while a strong positive relationship between IVD and both minor and major veins was detected. Notably, the vein density of the RDP1 sub-population tends to be negatively related to vein size, number of the mesophyll cell layer, and leaf thickness. Given our finding that high vein density positively relates to thin leaf and narrow veins which also correlate to a smaller veins space, it can be hypothesized that narrow or smaller leaf vein is a prerequisite for high vein density in rice leaves. An increase in vein density in rice leaves due to alteration in vein diameter and a change in mesophyll cell size or number were reported (Smillie et al., 2012; Feldman et al., 2014; Chatterjee et al., 2016).

**Figure 3:**
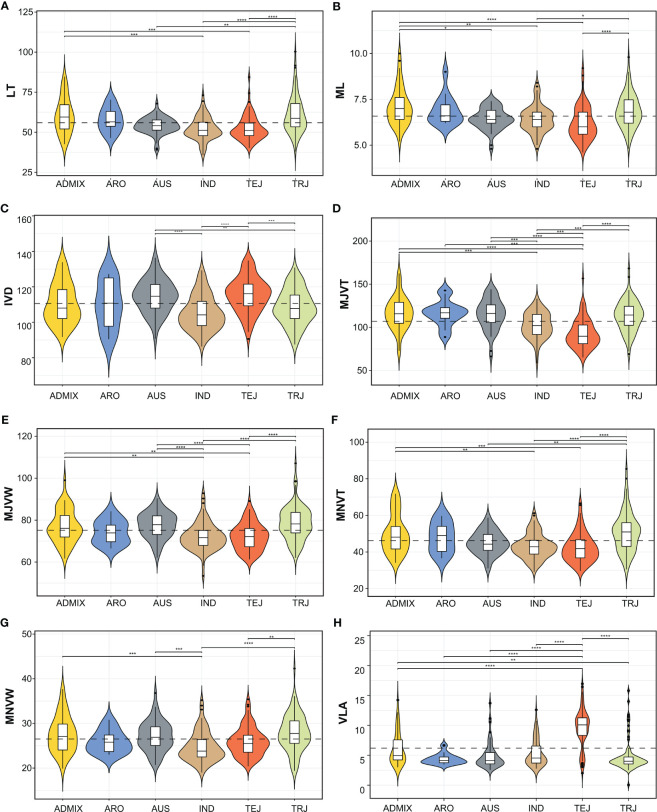
Violin plots of the distribution of leaf anatomical and vein traits. The dotted line represents the mean of each trait. **(A)** leaf thickness (LT), **(B)** number of mesophyll layers (ML), **(C)** inter-veinal distance (IVD), **(D)** major vein thickness (MJVT), **(E)** major vein width (MJVW), **(F)** minor vein thickness (MNVT), **(G)** minor vein width (MNVW), **(H)** vein length per leaf area (VLA). Asterisks indicate significant differences between sub-populations groups according to pairwise comparisons using Wilcoxon signed-rank test with Bonferroni correction (**¾ 0.001, ***< 0.0001, ****< 0.00001). ADMIX, admixture; ARO, aromatic; AUS, aus; IND, indica; TEJ, temperate japonica; TRJ, tropical japonica.

The RDP1 has five major sub-populations: i.e., indica (IND), aus (AUS), tropical japonica (TRJ), temperate japonica (TEJ), and aromatic (ARO) and admixture (ADMIX). Classification of LT according to sub-populations indicated significant differences between TRJ and TEJ while there was no significant difference in LT between TEJ and IND (**Figure 3A**). ADMIX, ARO, and TRJ had no significant differences in LT. For ML which showed an extremely positive relationship to LT, we observed a similar pattern of ML variation among the RDP1 sub-populations as in LT (**Figure 3B**). We found that the distances in between 2 adjacent minor veins of IND and TRJ were significantly lower than the other sub-population groups (**Figure 3C**). The variation in major vein; both MJVT and MJVW, of RDP1 observed in our work are well depicted in **Figures 3D, E**. The MJVT of TEJ was the lowest and significantly different from the others (**Figure 3D**). For the thickness of minor veins, TEJ together with IND were also lower than the other sub-population groups (**Figure 3F**), which is corresponded with the LT and ML results. TEJ and IND were also exhibited the significantly lower MNVW than the other groups as shown in (**Figure 3G**). When vein density was classified according to the sub-population groups, we found that the VLA of TEJ was extensively high and significantly different from all the other groups (**Figure 3H**). We observed that there was no statistically significant difference between TRJ, ARO, and ADMIX in all the traits. Here, the leaf shape of TRJ, ADMIX, and ARO sub-population tend to be thick leaves with thick and wide major and minor veins and low vein density.

### Multi-model GWAS analysis of leaf anatomical and vein traits

**Figure 4:**
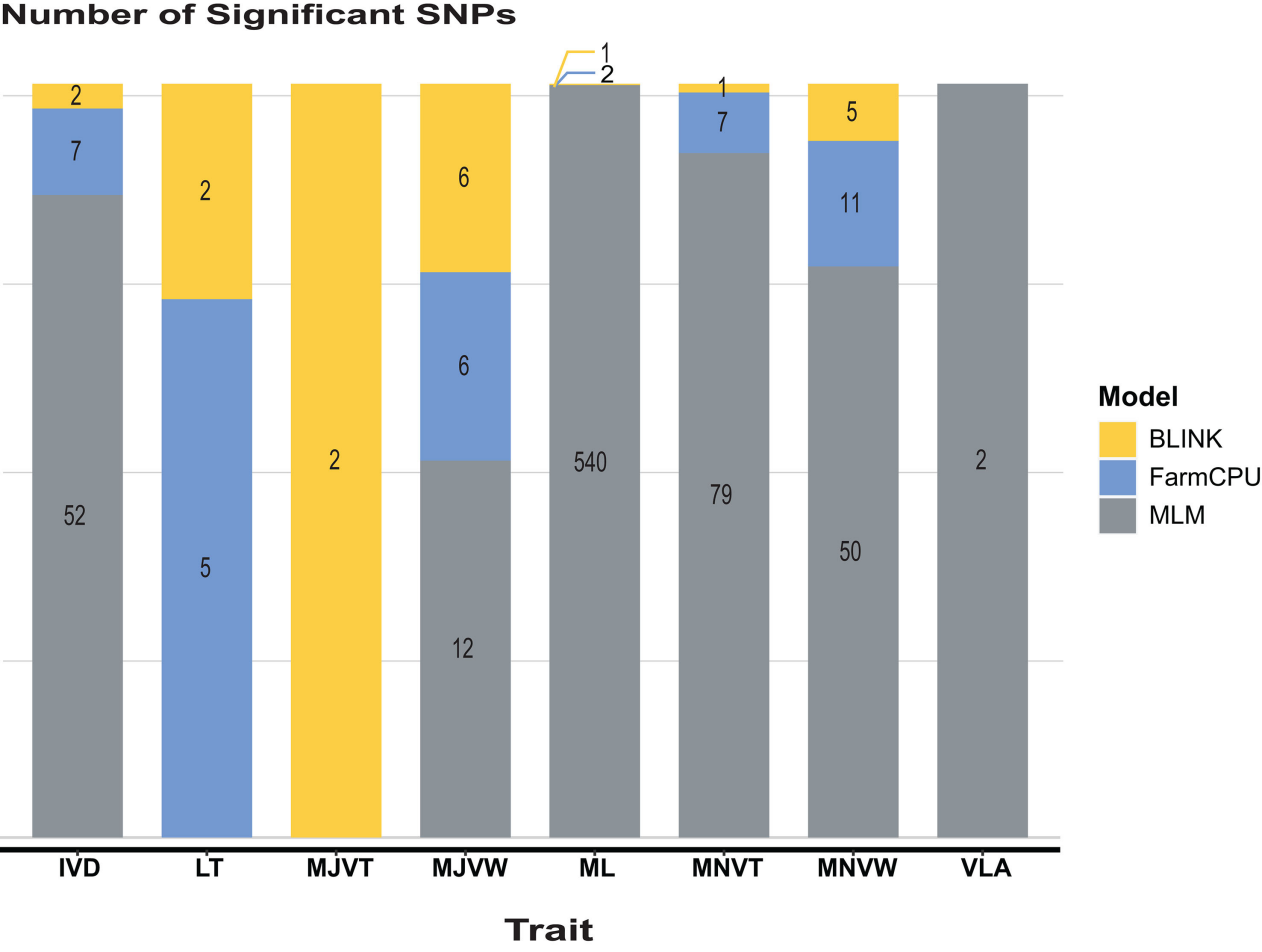
The number of significant SNPs identified within the RDP1. Models compared are Bayesian information and linkage-disequilibrium iteratively nested keyway (BLINK), fixed and random model circulating probability unification (FarmCPU), and mixed-linear model (MLM). IVD, inter-veinal distance; LT, leaf thickness; MJVT, major vein thickness; MJVW, major vein width; ML, number of mesophyll layers; MNVT, minor vein thickness; MNVW, minor vein width; VLA, vein length per leaf area.

GWAS analysis for leaf anatomical and vein traits in RDP1 using three GWAS models: BLINK, FarmCPU, and MLM, was performed by the R-based GAPIT program. In our study, a threshold of the genome-wide significance at 7 x 10^-8^ and a false discovery rate (FDR) cut-off with an alpha of 0.05 were determined. In almost all the traits, MLM, the only single locus method used here, predicted the highest number of significant single nucleotide polymorphisms (SNPs), especially in ML traits (**Figure 4**). The multi-locus models i.e., BLINK and FarmCPU, which are usually employed to reduce false positives in GWAS analysis implemented the highest number of significant SNPs in MJVT and LT, respectively. It is noted that there were no significant SNPs implemented by MLM in either LT or MJVT traits. Moreover, BLINK and MLM were the only models that identified the significant SNPs associated with MJVT and VLA, respectively.

**Figure 5:**
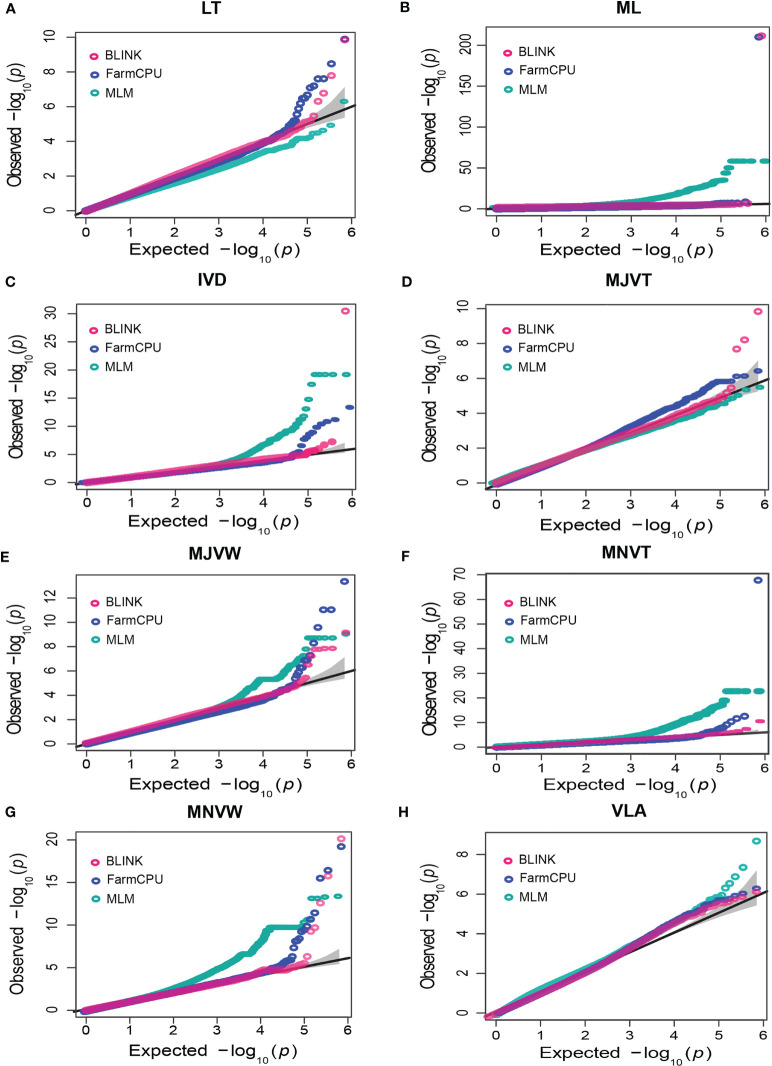
Quantile-quantile (QQ) plots of Bayesian information and linkage-disequilibrium iteratively nested keyway (BLINK), fixed and random model circulating probability unification (FarmCPU), and mixed-linear model (MLM) for leaf anatomical and vein traits. **(A)** leaf thickness (LT), **(B)** number of mesophyll layers (ML), **(C)** inter-veinal distance (IVD), **(D)** major vein thickness (MJVT), **(E)** major vein width (MJVW), **(F)** minor vein thickness (MNVT), **(G)** minor vein width (MNVW), **(H)** vein length per leaf area (VLA). The solid black line is the identity line showing the expected null distribution of the *P value* assuming no associations. The grey area represents the 95% concentration band.

According to the Q-Q plots, FarmCPU and BLINK models had a straight line with a sharp upward deviated tail which implied that both false positives and false negatives were efficiently controlled in almost all the traits, especially in LT and MNVW traits (**Figure 5**). However, for ML traits, the Q-Q plots for both models deflated downward and most of the SNPs were very close to the identity line reflecting that FarmCPU and BLINK may have been reported false negatives (**Figure 5B**). Notably, the FarmCPU exhibited strongly inflated *P* values in both ML and MNVT while the BLINK model inflated the *P* values in ML and IVD. Regarding ML, MNVT, and VLA which were the highest heritability traits here, the MLM model rather than FarmCPU and BLINK could effectively control both false positives and false negatives as indicated by the straight line with a slightly deviated tail of the Q-Q plots (**Figures 5B, H**). Q-Q plots of the FarmCPU model for LT, MJVW, MNVW, and IVD followed a straight line of the identity line with a sharp deviated tale implying that FarmCPU was the most powerful model in controlling both false positives and false negatives for the leaf anatomical and vein traits (**Figures 5A, C–G**). Interestingly, it was only the Q-Q plots of the BLINK model for MJVT which had a straight line close to the identity line with a sharp deviated tail compared to other models.

**Figure 6:**
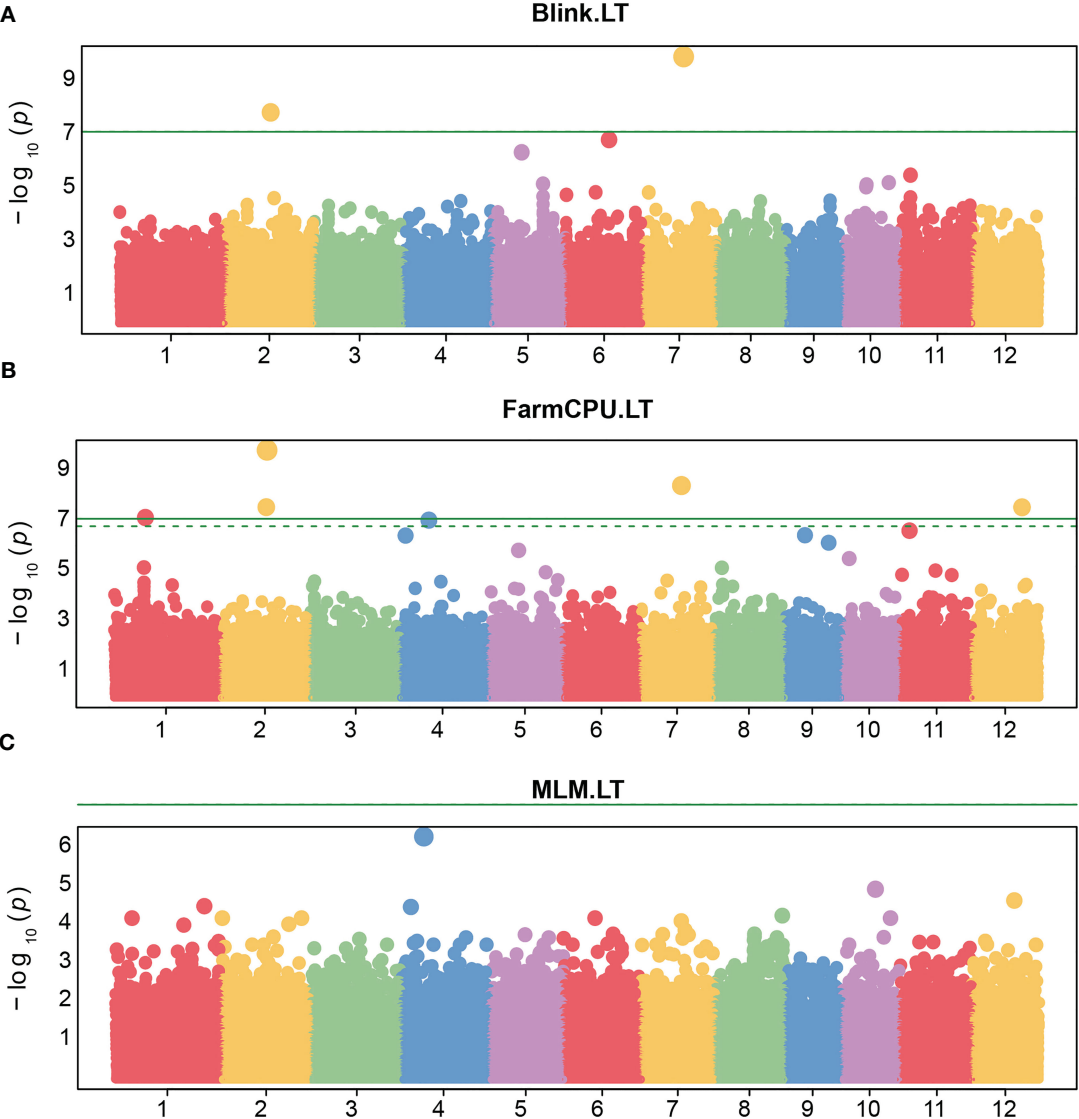
Manhattan plots of -log10 (*P* value) versus the physical location of SNPs across the 12 chromosomes associated with rice leaf thickness (LT) from the three models including Bayesian information and linkage-disequilibrium iteratively nested keyway (BLINK). **(A)**, fixed and random model circulating probability unification (FarmCPU) **(B)**, and mixed-linear model (MLM) **(C)**. The green horizontal line represents the genome-wide significance threshold of Bonferroni adjusted p-value = 7 × 10^−8^. The dotted line represents the threshold of false discovery rate adjusted p-value.

**Figure 7:**
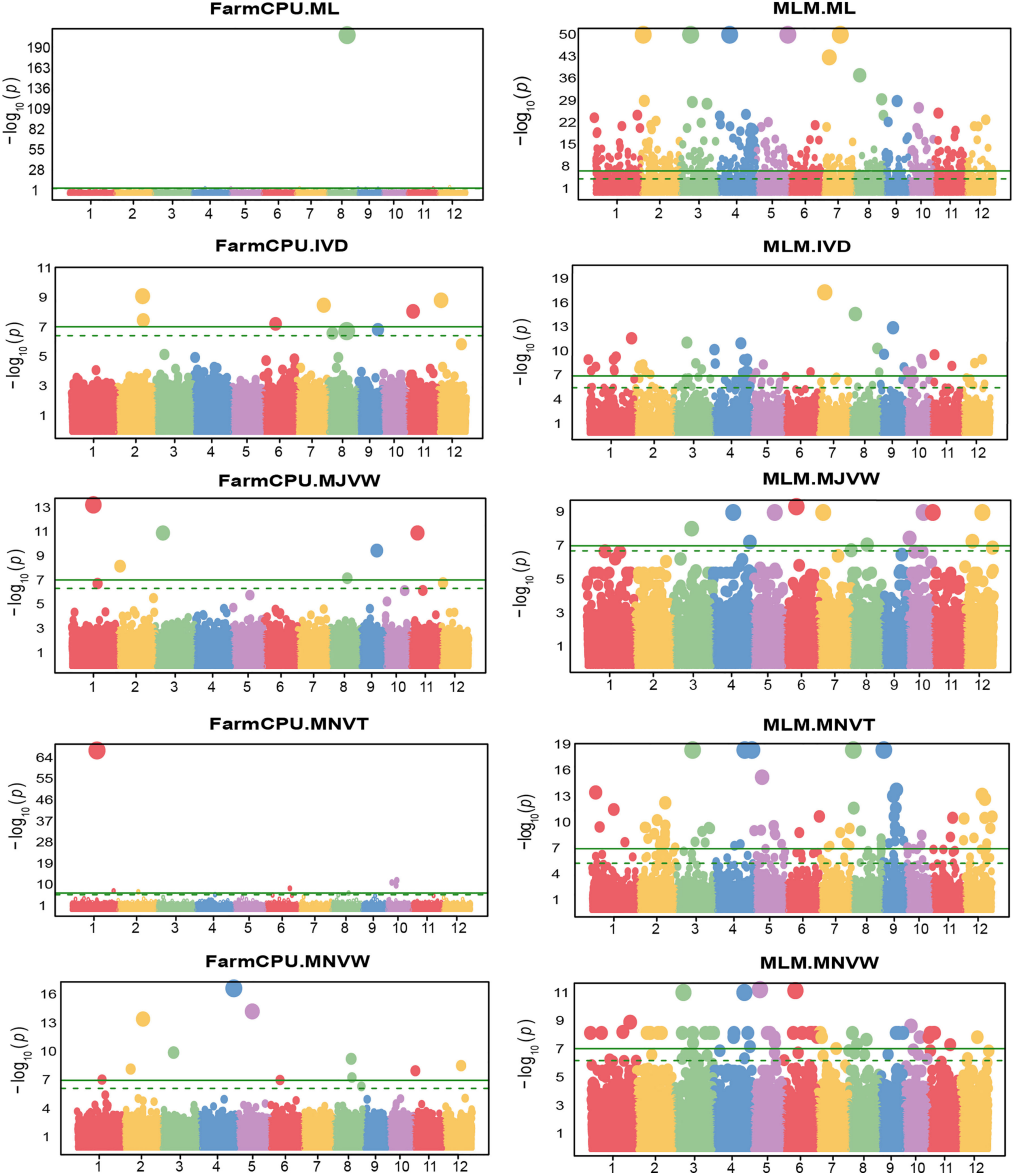
Manhattan plots of -log10 (*P* value) versus the physical location of SNPs across the 12 chromosomes associated with the number of mesophyll layer (ML), inter-veinal distance (IVD), major vein width (MJVW), minor vein thickness (MNVT), and minor vein width (MNVW) in rice from fixed and random model circulating probability unification (FarmCPU) and mixed-linear model (MLM). The green horizontal line represents the genome-wide significance threshold of Bonferroni adjusted p-value = 7 × 10^−8^. The dotted line represents the threshold of false discovery rate adjusted p-value.

Regarding the FarmCPU model, five SNPs were significantly associated with the leaf thickness trait in rice (**Figure 6**). Among them, there were two SNPs i.e, LOC_Os07g28280 and LOC_Os02g30730 located on chromosome 7 and chromosome 2 respectively, which were similar to the position of the only two significant SNPs associated with LT identified by the BLINK model. However, the FarmCPU and BLINK models identified the most significant SNPs associated with LT at different positions located on chromosome 2 and chromosome 7, respectively. As we observed the efficiency of the FarmCPU model and the fact that the FarmCPU and the BLINK models identified similar significant loci in many traits (**Table 2**), a comparison of the FarmCPU model with MLM for MJVW, MNVW, MNVT, and IVD traits were performed (**Figure 7**). For these traits, the FarmCPU model identified single significant SNPs with higher *P* values and more precise chromosome positions. MLM showed the lower significant SNPs *P* values with broader peaks which were containing multiple significant SNPs or SNP clusters in Manhattan plots. Therefore, the number of the significant SNPs identified with the MLM model was larger than with the FarmCPU model. Moreover, the identified significant SNPs from the MLM were different from the position of the significant SNPs identified by the FarmCPU model which strongly inflated the *P* value. The inflation of the *P* values implemented by the FarmCPU resulted in a lower number of identified SNPs with the *P* values smaller than the threshold. We noticed that some of the significant SNPs identified by the MLM model were among the non-significant SNPs implemented by the FarmCPU model.

**Table 2:** Candidate genes for each significant SNP associated with leaf anatomical and vein traits implemented by BLINK and FarmCPU models.

Trait	Model	SNP ID	Position	Alleles	Chr	*P*-Value	Locus ID	Associated Gene	Known Effect on Leaf Tissue
**LT**	BLINK	7.16541764	16542758	A/G	7	1.13E-10	LOC_Os07g28280	SLG	Control leaf angle/brassinosteroid homeostasis (Feng et al., 2016)
	BLINK	2.18295322	18301192	G/A	2	1.35E-08	LOC_Os02g30730	DOT2	Vascular development (Petricka et al., 2008)
	FarmCPU	2.18614125	18619995	A/G	2	1.31E-10	LOC_Os02g31140	CFL1	Leaf cuticle development (Wu et al., 2011)
	FarmCPU	7.16541764	16542758	A/G	7	3.38E-09	LOC_Os07g28280	SLG	
	FarmCPU	2.18295322	18301192	G/A	2	2.46E-08	LOC_Os02g30730	DOT2	
	FarmCPU	1.12875021	12876048	A/G	1	6.4E-08	LOC_Os01g22910	GA2OX2	Gibberellin metabolic process/control plant height (Zhou et al., 2015)
**ML**	BLINK	8.18919729	18922443	C/T	8	5.1E-276	LOC_Os08g30740	ABCA3	
	FarmCPU	8.18919729	18922443	C/T	8	1.0E-210	LOC_Os08g30740	ABCA3	
	FarmCPU	12.11231292	11233957	A/G	12	4.01E-09	LOC_Os12g19381	RBCS3	
**IVD**	BLINK	8.18919729	18922443	C/T	8	3.39E-31	LOC_Os08g30740	ABCA3	
	BLINK	3.31061057	31068171	T/C	3	5.63E-08	LOC_Os03g54160	RAP1B	
	FarmCPU	8.18919729	18922443	C/T	8	1.79E-11	LOC_Os08g30740	ABCA3	
	FarmCPU	2.24096070	24101940	G/C	2	5.95E-10	LOC_Os02g39920	BIP135	
	FarmCPU	12.3194875	3195873	C/T	12	1.14E-09	LOC_Os12g06520	RSG	Gibberellin metabolic process/plant height (Phanchaisri et al., 2012)
	FarmCPU	7.25919115	25920110	G/T	7	2.46E-09	LOC_Os07g43360	HAM701	
	FarmCPU	11.5805479	5809728	A/T	11	6.41E-09	LOC_Os11g10590	DT11	Stomatal density (Li et al., 2017)
	FarmCPU	2.24733354	24739224	A/G	2	2.54E-08	LOC_Os02g40860	HBD2	Auxin sensitivity/control leaf angle (Duan et al., 2006)
	FarmCPU	6.11734343	11735343	C/G	6	4.52E-08	LOC_Os06g20410	PHD27	
**MJVT**	BLINK	8.21350927	21353641	A/G	8	1.45E-10	LOC_Os08g34010	ZF-HD homeobox	
	BLINK	9.9652726	9653728	C/T	9	6.15E-09	LOC_Os09g15770	BIP107	
	BLINK	2.14209586	14215457	A/G	2	2.07E-08	LOC_Os02g24430	SNDP2	
**MJVW**	BLINK	3.6927409	6928412	G/A	3	5.96E-10	LOC_Os03g12860	HOX19	
	BLINK	11.7727019	7731275	A/G	11	1.31E-08	LOC_Os11g13930	OsZHD4	
	BLINK	8.16537239	16539954	T/G	8	1.33E-08	LOC_Os08g26990	RR13	Cytokinin metabolism/plant height (Hirose et al., 2007)
	BLINK	4.152230	153231	G/A	4	1.55E-08	LOC_Os04g01160	SOR1	
	BLINK	1.21020604	21021650	C/T	1	1.57E-08	LOC_Os01g37670	OsFbox018	
	FarmCPU	1.21020604	21021650	C/T	1	4.37E-14	LOC_Os01g37670	OsFbox018	
	FarmCPU	11.7727019	7731275	A/G	11	9.21E-12	LOC_Os11g13930	OsZHD4	
	FarmCPU	3.6927409	6928412	G/A	3	9.31E-12	LOC_Os03g12860	HOX19	
	FarmCPU	9.15858193	15859195	G/A	9	2.64E-10	LOC_Os09g26210	DLN224/	
	FarmCPU	2.2748749	2748752	A/T	2	5.12E-09	LOC_Os02g05640	HOX26	
**MNVT**	BLINK	6.17581416	17582414	G/A	6	4.43E-09	LOC_Os06g30440	GH3-7	Response to auxin stimulus (Jain et al., 2006)
	FarmCPU	1.24000718	24001763	A/C	1	1.62E-68	LOC_Os01g42294	OsRPK1	
	FarmCPU	10.5478731	5462195	G/A	10	2.38E-12	LOC_Os10g10040	Cytochrome P450	
	FarmCPU	1.39556456	39557500	C/T	1	9.78E-09	LOC_Os01g68000	PLA2	Cell division/Control leaf width/leaf length/leaf shape (Mimura et al., 2012)
**MNVW**	BLINK	3.6927409	6928412	G/A	3	7.31E-21	LOC_Os03g12860	HOX19	
	BLINK	3.12061864	12063147	C/G	3	1.71E-16	LOC_Os03g21210	CEL9D	Cell elongation/Cell wall organization (Zhou et al., 2006)
	BLINK	12.16416578	16419286	A/T	12	2.54E-13	LOC_Os12g27810	OsFbox650	
	BLINK	2.2748749	2748752	A/T	2	1.99E-10	LOC_Os02g05640	HOX26	
	BLINK	2.7578678	7578679	G/T	2	5.5E-10	LOC_Os02g13900	BZR4	Brassinosteroid signaling (Bai et al., 2007)
	FarmCPU	4.32197314	32382425	G/A	4	1.46E-17	LOC_Os04g54340	MRE11	Cell division/growth and development (Shen et al., 2020)
	FarmCPU	5.14240577	14298035	T/A	5	3.93E-15	–	IPT3	Cytokinin biosynthetic process (Sakamoto et al., 2006)
	FarmCPU	2.19228551	19234420	G/T	2	2.49E-14	LOC_Os02g32520	ERD1	
	FarmCPU	3.12061864	12063147	C/G	3	8.48E-11	LOC_Os03g21210	CEL9D	
	FarmCPU	12.16416578	16419286	A/T	12	2.01E-09	LOC_Os12g27810	OsFbox650	
	FarmCPU	2.7578678	7578679	G/T	2	4.61E-09	LOC_Os02g13900	BZR4	
	FarmCPU	11.2381536	2385679	T/G	11	6.98E-09	LOC_Os11g05320	PIDL1	Auxin sensitivity/control leaf shape (Zhang et al., 2018)

Chr, Chromosome.

### Identification of candidate genes for the leaf anatomical traits

Candidate genes containing SNPs significantly associated with rice leaf anatomical traits were identified by the gene annotation network RGAP (http://rice.uga.edu/, accessed on 15 January 2022) and the Oryzabase database (Kurata and Yamazaki, 2006). Association analysis using the FarmCPU model identified five significant SNPs associated with LT traits. Among these, the most significant SNP was located on chromosome 2 near *CURLY FLAG LEAF 1* (*CFL1*) which encodes a WW domain protein regulating the cuticle development of leaf epidermal cells (Wu et al., 2011). The second most significant SNP detected by the FarmCPU, which was repeatedly identified by BLINK as the most significant SNP associated with LT, was located near *SLENDER GRAIN* (*SLG*) (**Table 2**). *SLG* regulates leaf angle and plant height in rice through cell division and expansion which is mediated by brassinosteroid signaling (Feng et al., 2016). A significant SNP located near *DEFECTIVELY ORGANIZED TRIBUTARIES 2* (DOT2) was identified by both the FarmCPU and BLINK models. In *Arabidopsis thaliana*, DOT2 is encoded by *At5g16780*, which corresponds to an 820-aa leucine zipper protein. *DOT2* mutation leads to disruption of vein patterning and an increase in leaf cell number together with a decrease in cell size (Petricka et al., 2008). An SNP on chromosome 1 positioned near *GIBBERELLIN 2-OXIDASE 2* (*GA2OX2*), which is a member of rice gibberellic acid (GA) 2-oxidase family genes, was identified by the FarmCPU model.

For the ML trait, FarmCPU and BLINK implemented 2 and 1 significant SNPs respectively. The significant SNP near *ABC TRANSPORTER A FAMILY MEMBER 3* (*ABCA3*) gene was repeatedly identified by both multi-locus models. In contrast, MLM identified 540 significant SNPs associated with ML that most of the SNP loci associated with known genes functioning in rice leaf development (**Table 3**). Intriguingly, three non-synonymous SNPs were located within the region of *NARROW LEAF 1* (*NAL1*) which regulates rice leaf shape, leaf width, and vein patterning *via* cell division and expansion (Zhang et al., 2014; Jiang et al., 2015; Taguchi et al., 2015; Lin et al., 2019). Moreover, another gene that regulates rice leaf width i.e., *NARROW LEAF 7* (*NAL7*) was associated with the significant SNP located on chromosome 3. *NAL7* encodes a flavin monooxygenase protein which plays an important role in regulating leaf width, bulliform cell, and vascular bundle development through the auxin signaling pathway (Fujino et al., 2008). We also found a significant SNP in *WUSCHEL-RELATED HOMEOBOX4* (WOX4) associated with the ML trait. *WOX*4 is a transcription factor promoting cell division thus controlling vascular bundle development and leaf width (Ohmori et al., 2013; Yasui et al., 2018). One of the most important candidate genes identified to be associated with this mesophyll cells trait was located on chromosome 4 near *ENT-KAURENE SYNTHASE 2* (*KS2*) gene which encodes a GA metabolic enzyme regulating mesophyll cells development in rice leaf (Ji et al., 2014). The significant locus on chromosome 7 is located within the region of *Ghd7.1* or *HD2* (*Heading date2*) which is recently reported as a key gene in the regulation of flag leaf size (Tang et al., 2018). Some of the significant SNPs associated with the number of mesophyll layer trait were in the candidate regions near the reported genes regulating cell division and cell expansion during leaf morphogenesis such as *VIRESCENT-ALBINO LEAF1* (*VAL1*) (Zhang T. et al., 2018), *Loose Plant Architecture1* (*LPA1*) (Wu et al., 2012), *RICE MINUTE-LIKE1* (*RML1*) (Zheng et al., 2016), and *CYSTEINE ENDOPEPTIDASE REP-2* or *VPE3* (Lu et al., 2016).

**Table 3:** Selected candidate genes with known effects on the leaf for each significant SNP associated with the number of mesophyll cells layer (ML) and interveinal distance (IVD) traits implemented by the MLM model.

Trait	SNP ID	Position	Alleles	Chr	*P*-Value	Locus ID	Associated Gene	Known Effect on Leaf Tissue
**ML**	3.10538437	10539522	G/A	3	3.45E-51	LOC_Os03g18820	OsXXT1	Cell wall organization (Wang et al., 2014)
	7.16944507	16945501	G/C	7	3.45E-51	LOC_Os07g28890	NRP1	Control leaf photosynthesis/biomass (Chen et al., 2021)
	8.5322896	5323894	G/A	8	2.72E-38	LOC_Os08g09210	VAL1	Cell division and leaf width (Zhang et al., 2018)
	3.12091545	12092828	G/T	3	8.86E-30	LOC_Os03g21210	CEL9D	Cell wall organization/cell elongation (Zhou et al., 2006)
	1.40090099	40091143	G/T	1	1.34E-25	LOC_Os01g69070	PIN5A	Auxin-mediated signaling pathway (Paponov et al., 2005)
	8.27399276	27401991	C/T	8	1.44E-25	LOC_Os08g43390	CYP78A15	Regulates leaf elongation rate (Maeda et al., 2019)
	12.1910112	19129685	G/A	12	3.92E-24	LOC_Os12g31810	CYCA2;1	cell division/cell differentiation/stomatal frequency/plant height (Qu et al., 2018)
	3.7210439	7211442	C/T	3	1.73E-21	LOC_Os03g13400	LPA1	Cell division and cell expansion (Wu et al., 2012)
	10.8908264	8979404	C/T	10	2.45E-20	LOC_Os10g17770	LHP1	Regulates leaf size/length/width and number (Gaudin et al., 2001)
	9.21884581	21885063	A/G	9	1.07E-18	LOC_Os09g38000	NAC109	Unidimensional growth (Li et al., 2017)
	4.30878337	31063452	G/C	4	1.38E-18	LOC_Os04g52240	KS2	Mesophyll cell development (Ji et al., 2014)
	6.28205663	28206662	G/A	6	1.19E-17	LOC_Os06g46410	ARF17	Control flag leaf angle (Huang et al., 2021)
	4.28257963	28443110	C/A	4	4.73E-16	LOC_Os04g47870	AGO1b	Sclerenchyma cell development (Li et al., 2019)
	1.2423264	24233685	C/G	1	1.19E-15	–	RAN1	Growth and development (Xu and Cai, 2014)
	4.31016488	31201599	G/A	4	2.15E-15	LOC_Os04g52479	NAL1	Cell division, cell expansion/ Control leaf shape/leaf width/vascular development (Zhang et al., 2014; Lin et al., 2019)
	4.30979400	31164510	G/T	4	6.68E-15	LOC_Os04g52479	NAL1	
	4.30975553	31160663	C/T	4	5.14E-08	LOC_Os04g52479	NAL1	
	1.6979217	6980218	A/G	1	6.16E-15	LOC_Os01g12690	OFP1	Control leaf angle (Xiao et al., 2017)
	4.32935165	33120277	G/A	4	8.65E-13	LOC_Os04g55590	WOX4	Cell division and vascular differentiation/leaf width (Ohmori et al., 2013; Yasui et al., 2018)
	7.9168132	9169127	T/A	7	2.89E-12	LOC_Os07g15770	GHD7	Control plant height/heading date/yield potential (Xue et al., 2008)
	8.19469252	19471966	C/T	8	3.30E-11	LOC_Os08g31470	PAY1	Control plant height/stem thickness and vascular bundle number (Zhao et al., 2015)
	12.1064089	1065090	C/T	12	6.55E-11	LOC_Os12g02870	SCR	Asymmetric cell division/stomatal development (Hughes and Langdale, 2022)
	12.1062017	1063018	G/A	12	2.3E-09	LOC_Os12g02870	SCR	
	3.3400715	3401720	A/G	3	4.25E-10	LOC_Os03g06654	NAL7	Bulliform cell and vascular development/Control the development of leaf width (Fujino et al., 2008)
	2.2232036	22326230	G/T	2	9.79E-10	LOC_Os02g36974	GID2	GA sensitivity/Control leaf width (Liu et al., 2016)
	9.13741161	13742163	G/A	9	1.22E-09	LOC_Os09g23200	SLL1	Leaf abaxial cell development/leaf rolling
	9.13779603	13780605	G/C	9	2.63E-08	LOC_Os09g23200	SLL1	(Zhang et al., 2009)
	11.3314467	3318565	T/C	11	3.42E-09	LOC_Os11g06750	RML1	Cell expansion/Regulates leaf morphology and plant architecture (Zheng et al., 2016)
	7.24193662	24194657	G/A	7	9.19E-09	LOC_Os07g40300	ZFP7	Leaf morphogenesis (Liu et al., 2018)
	4.28466414	28651564	G/A	4	2.07E-08	LOC_Os04g48070	ROC4	Control leaf shape/wax biosynthesis (Guo et al., 2019)
	2.21691409	21697279	G/A	2	2.32E-08	–	MIR1848	Wax biosynthesis/leaf angle (Xia et al., 2015)
	7.29659723	29660716	G/A	7	2.62E-08	LOC_Os07g49460	Ghd7.1	Control flag leaf size (Tang et al., 2018)
	12.26455856	26489463	G/A	12	2.69E-08	LOC_Os12g42610	YAB6	Bulliform cell development (Xia et al., 2017)
	2.25852239	25858109	G/T	2	9.34E-08	LOC_Os02g43010	VPE3	Cell expansion (Lu et al., 2016)
**IVD**	7.16944507	16945501	G/C	7	5.26E-20	LOC_Os07g28890	NRP1	
	8.5322896	5323894	G/A	8	1.49E-15	LOC_Os08g09210	VAL1	
	1.40090099	40091143	G/T	1	1.53E-12	LOC_Os01g69070	PIN5A	
	3.12091545	12092828	G/T	3	5.16E-12	LOC_Os03g21210	CEL9D	
	12.1910112	19129685	A/T	12	6.19E-10	LOC_Os12g31810	CYCA2;1	
	4.30878337	31063452	G/C	4	1.09E-09	LOC_Os04g52240	KS2	
	4.32935165	33120277	G/A	4	2.21E-08	LOC_Os04g55590	WOX4	
	4.28257963	28443110	C/A	4	7.85E-08	LOC_Os04g47870	AGO1b	
	4.309794	31164510	G/T	4	9.12E-08	LOC_Os04g52479	NAL1	

Chr, Chromosome.

Regarding the significant SNPs associated with the space in between minor veins or IVD trait, we observed some of the significant SNPs which were repeatedly detected in the ML trait by all the models (**Table 3**). For instance, the *ABCA3* gene was identified as the most significant SNP associated with IVD by FarmCPU and BLINK. *NAL1*, *WOX4*, and *VAL1*, which regulate rice leaf width and vascular bundle development, were also repeatedly identified by MLM as the candidate genes for the IVD trait. We noticed that *CELLULASE 9D* (CEL9D) was repeatedly detected as a candidate locus associated with ML, IVD, MNVT, and MNVW by FarmCPU, BLINK, and MLM models.

### Identification of candidate genes for the vein traits

Candidate genes containing SNPs associated with the five vein traits including MJVT, MJVW, MNVT, MNVW, and VLA, were identified. Regarding MJVT, BLINK was the only model here that could identify some significant SNPs for this vein trait (**Table 2**). The most significant SNP associated with the thickness of the major vein trait was LOC_Os08g34010 which encodes for a zinc finger homeodomain protein. The second most significant locus for MJVT was LOC_Os09g15770 encoding for a brassinosteroid receptor kinase (BRI1)-interacting protein 107. For the width of the major vein trait, MLM identified the most significant SNP near *DWARF53* (*D53*) on chromosome 11 (**Table 4**). The *D53* gene encodes for a class I Clp ATPase protein which is a repressor for the strigolactones hormone signaling pathway. Mutation of *D53* leads to alteration in both small and large vascular bundle numbers in rice internode as well as a reduction in leaf length (Zhou et al., 2013). The SNP locus on chromosome 4, LOC_Os04g56620, which was detected by MLM as a candidate gene for MJVW, was located within the interval of gene *CO-FACTOR FOR NITRATE REDUCTASE AND XANTHINE DEHYDROGENASE 1* (*CNX1*) whose functional annotation is nitrate reductase and xanthine dehydrogenase co-factor. Slender and twisted leaves were observed in *CNX1* mutant rice plants (Liu et al., 2019). The peak on chromosome 8 near *MEDIATOR 14_1* (*MED14_1*) was one of the significant loci of MJVW. The *MED14_1* is an RNA polymerase II transcription cofactor that plays an important role in vascular bundle development. This gene functions in cell division and differentiation and thus regulates a number of both minor and major veins and rice leaf width (Malik et al., 2020). The *Arabidopsis* Mediator gene *STRUWWELPETER* (*SWP*) encodes subunits of the Mediator transcriptional regulatory complex of RNA polymerase II activity. The *swp* mutant reduced cell numbers of leaf primordium which leads to small leaves with aberrant morphology (Autran et al., 2002).

**Table 4:** Top candidate genes for each significant SNP associated with vein traits implemented by the MLM model.

Trait	SNP ID	Position	Alleles	Chr	P-Value	Locus ID	Associated Gene	Known Effect on Leaf Tissue
**MJVW**	11.144973	145973	C/A	11	7.44E-10	LOC_Os11g01330	D53	Control strigolactone signaling/leaf length/vascular bundle number (Zhou et al., 2013)
	12.16516462	16519170	C/T	12	7.44E-10	LOC_Os12g27994	DEC	Control phyllotactic patterning *via* cytokinin signaling pathway (Itoh et al., 2012)
	4.33577355	33762476	C/T	4	4.24E-08	LOC_Os04g56620	CNX1	Control leaf width/leaf shape (Liu et al., 2019)
	8.14731173	14733888	G/A	8	6.07E-08	LOC_Os08g24400	MED14_1	Control cell division/leaf width and vascular bundle development (Malik et al., 2020)
**MNVT**	4.33563482	33748603	G/A	4	2.77E-19	LOC_Os04g56620	CNX1	
	4.26899384	27084513	G/A	4	2.77E-19	LOC_Os04g45810	HOX22	
	9.1370533	1371534	G/A	9	2.77E-19	LOC_Os09g02830	MADS78	
	5.7705398	7705458	T/G	5	3.89E-16	LOC_Os05g13900	OsPRP	
	1.4281937	4282938	T/A	1	2.24E-14	LOC_Os01g08700	GI	
	12.17354735	17360781	C/T	12	4.13E-14	LOC_Os12g29330	NAC139	Cell wall organization/xylem development (Shen et al., 2009)
	9.12506545	12507547	G/A	9	1.41E-12	LOC_Os09g20820	Eno1	
	6.30959662	30960661	C/T	6	1.28E-11	LOC_Os06g51110	CYCB2;2	Cell division/gibberellic acid sensitivity (Sauter, 1997)
	6.30849308	30850307	T/C	6	1.38E-11	LOC_Os06g50920	ILA1	Control leaf angle through vascular bundle size and sclerenchyma cell number (Ning et al., 2011)
	2.7739591	7739593	C/T	2	2.57E-10	LOC_Os02g14130	GSK3	Brassinosteroid-mediated signaling (Gao et al., 2019)
	8.27272018	27274733	G/A	8	4.86E-09	LOC_Os08g43130	LPL3	Control of leaf epidermal cell morphogenesis (Zhou et al., 2016; Huang et al., 2019)
	2.9511306	9511309	G/A	2	5.01E-09	LOC_Os02g16730	EXPA13	Cell wall organization (Lee et al., 2001)
	2.20938827	20944696	G/A	2	8.69E-09	LOC_Os02g34884	GH1	Regulates cell growth and development (Guo et al., 2020)
	9.19758053	19758535	T/C	9	8.69E-09	LOC_Os09g33490	ONAC1	Candidate gene associated with flag leaf thickness (Chen et al., 2022)
	9.11881266	11882268	G/A	9	9.86E-09	LOC_Os09g19930	ONI3	Leaf primordium development/leaf length (Fang et al., 2015)
	3.17297698	17298889	C/T	3	1.13E-08	LOC_Os03g30250	BC1	Secondary wall formation/Control cell wall thickness of sclerenchyma cell and vascular bundle (Li et al., 2003)
	1.31420768	31421813	T/C	1	1.26E-08	LOC_Os01g54620	BC7	Secondary wall formation (Wei et al., 2008)
	3.26726269	26733216	G/A	3	1.39E-08	LOC_Os03g47230	PSK5	Cell differentiation (Lorbiecke and Sauter, 1999)
	2.34787197	34793067	C/T	2	5.06E-08	LOC_Os02g56760	OsFbox116	Control flag leaf width (Du et al., 2022)
**MNVW**	5.7632474	7632534	C/T	5	4.61E-12	LOC_Os05g13790	CMT3	
	3.7951604	7952667	C/T	3	7.42E-12	LOC_Os03g14669	HAP5C	
	4.28385427	28570577	C/A	4	7.42E-12	LOC_Os04g48070	ROC4	
	1.3731599	37317034	T/A	1	9.37E-10	–	REL1	Control of leaf rolling and bending (Chen et al., 2015)
	11.140467	1405670	C/T	11	5.44E-09	LOC_Os11g03540	WRI1-1	
	11.4373881	4377980	C/T	11	5.44E-09	LOC_Os11g08340	GH3-12	Auxin sensitivity (Jain et al., 2006)
	2.12511053	12511058	C/T	2	5.44E-09	LOC_Os02g21090	bHLH139	
	2.9878212	9878215	C/T	2	5.44E-09	LOC_Os02g17230	YUCCA12	Control leaf shape (Fujino et al., 2008)
	8.3320029	3321027	C/T	8	5.44E-09	LOC_Os08g06100	ROMT9	Control flag leaf width/vascular bundle size and number (Huangfu et al., 2022)
	3.36125171	36132246	G/T	3	5.44E-09	LOC_Os03g63970	GNP1	Cell elongation/Gibberellin metabolic process (Tong et al., 2014)
	7.2805349	2806349	G/A	7	5.44E-09	LOC_Os07g05900	PROG1	
	12.16516462	16519170	C/T	12	1.09E-08	LOC_Os12g27994	DEC	
	11.19684879	20150958	A/C	11	3.72E-08	LOC_Os11g34300	MRG702	Control of leaf rolling and bending/flag leaf length (Jin et al., 2015)
**VLA**	2.10015824	10015827	G/A	2	1.74E-09	LOC_Os02g17390	AIM1	Cell wall organization/Salicylic acid biosynthesis (Richmond and Bleecker, 1999; Xu et al., 2017)
	11.6598118	6602366	G/C	11	3.72E-08	LOC_Os11g11960	–	

Chr, Chromosome.

The only significant locus of MNVT identified by BLINK was located near *GH3-7* which is a member of the auxin-responsive GH3 gene family in rice (Jain et al., 2006). The FarmCPU detected significant SNPs on chromosome 1 near *PLASTOCHRON 2* (*PLA2*) whose function in rice leaf development is well documented. The *PLA2* controls the cell cycle and vegetative growth time during rice leaf morphogenesis and regulates rice leaf shape, width, and length (Mimura et al., 2012). Regarding the MLM model, a higher number of significant loci for MNVT were identified. Among these, a peak on chromosome 6 located in the interval of *INCREASED LEAF ANGLE 1* (*ILA1*) was detected. *ILA1* functions in secondary cell wall biogenesis and plays a major role in controlling leaf inclination through the regulation of vascular bundle size and sclerenchymatous cell number (Ning et al., 2011). A significant SNP on chromosome 9 was associated with *ONION3* (*ONI3*) which is important for leaf primordium development and regulation of rice leaves (Fang et al., 2015). Interestingly, two significant loci related to known genes regulating secondary wall formation and cell wall thickness of vascular bundles, *BRITTLE CULM 1* (*BC1*) (Li et al., 2003) and *BRITTLE CULM 7* (*BC7*) (Wei et al., 2008), were identified for MNVT by MLM. Moreover, LOC_Os02g56760 encodes for F-box protein 116 which is reported as a candidate gene in controlling flag leaf width in rice (Du et al., 2022) was also identified as a significant SNP here. Intriguingly, MLM analysis for MNVT also reported a significant SNP on chromosome 9 located within the interval of the *qFTL9* which is reported as a QTL associated with rice leaf thickness (Chen et al., 2022).

For MNVW, multiple significant SNPs identified by FarmCPU were similar to the significant loci identified by BLINK (**Table 2**). For example, the peak on chromosome 2 near *BRASSINAZOLE RESISTANT 4* (*BZR4*) plays an important role in brassinosteroid signaling transduction (Bai et al., 2007). The distinct significant SNP, identified by only the FarnCPU model, was located in the interval of *MEIOTIC RECOMBINATION 11* (*MRE11*) which is essential for the regulation of the cell cycle and required for normal vegetative growth and development in rice (Shen et al., 2020). The MLM model identified 50 significant SNPs in association with the MNVW trait. Among these, the most significant SNP was located near *RICE OUTMOST CELL-SPECIFIC GENE 4* (*ROC4*). *ROC4* is a GLABRA2-type homeobox gene that regulates leaf cuticular wax development (Wang et al., 2018). Up-regulation of the *ROC4* gene in the rice *Oschr4-5* mutant which produces narrow and rolled leaves with reduced vascular bundle number was reported (Guo et al., 2019). Moreover, the peak on chromosome 1 was located within the interval of *ROLLED AND ERECT LEAF 1* (*REL 1*) which plays a crucial role in leaf rolling and bending. Leaves of the *rel1* mutant are rolled and reduced in natural width due to an increase in the adaxial bulliform cell numbers and size (Chen et al., 2015). MLM also identified a significant locus related to another gene known in the regulation of leaf rolling and bending, *MORF-RELATED GENE702* (*MRG702*) which encodes a reader protein for brassinosteroid (BR)-related genes (Jin et al., 2015). Strikingly, the significant SNP on chromosome 8 was located near *ROMT9* (*OsCOMT*) which is recently reported as a key gene in the regulation of flag leaf width, vascular bundle size, and number (Huangfu et al., 2022).

Regarding the vein density trait VLA, the MLM was the only model that implemented two significant SNPs in this study. The most significant locus *LOC_ Os02g17390* was located in the interval of *ABNORMAL INFLORESCENCE MERISTEM 1* (*AIM1*) which functions in cell wall organization and salicylic acid biosynthesis (Richmond and Bleecker, 1999; Xu et al., 2017). The second most significant locus associated with VLA was near LOC_Os11g11960 which encodes for an NB-ARC domain-containing protein.

### Haplotype analysis of the candidate genes

**Figure 8:**
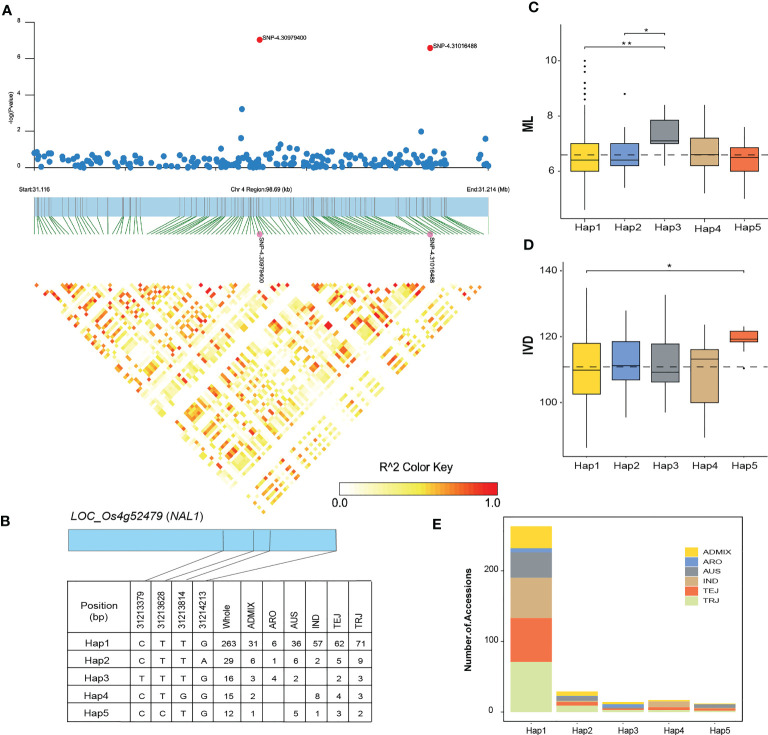
Haplotype analysis of *LOC_Os04g52479* (*NAL1*). **(A)** Local Manhattan plot (top) and LD heatmap (bottom) of a haplotype block on chromosome 4 associated with the number of mesophyll layer (ML) and inter-veinal distance (IVD) in RDP1 sub-population. The red dots indicate the lead SNP 4.31016488 and SNP 4.30979400. **(B)** Haplotypes of *LOC_Os04g52479*. **(C)** The distribution of ML and **(D)** IVD in the RDP1 sub-population for the five haplotypes of *LOC_Os04g52479*. Asterisk indicates significant differences among haplotypes according to the Kruskal-Wallis test and the pairwise Wilcoxon test (*P*< 0.05). **(E)** Frequency of the five haplotypes of *LOC_Os04g52479* in the RDP1 sub-population. The black dot indicates the outlier data.

We selected 4 candidate genes at 4 loci and performed a haplotype analysis to detect significant differences in ML, IVD, MNVW, MJVW, and VLA traits between different haplotypes of each gene. For *LOC_Os04g52479* (*NAL1*) on chromosome 4, the LD block region was started from 31.116 to 31.214 Mb (96.69 kb) and included 6 SNPs (**Figure 8A**). Five major haplotypes of *LOC_Os04g52479* were detected in the region shared by at least 10 accessions of the RDP1 (**Figure 8B**). Hap1 is prevalent in the whole RDP1 population, especially in the TRJ sub-population. Hap3 had a significantly higher mean ML than Hap1 and Hap2 (**Figure 8C**). However, Hap1 is predominant in the accessions which showed a higher ML. *LOC_Os04g52479* was also selected for haplotype analysis for IVD. In a comparison of IVD across the five haplotypes, Hap5 had a significantly higher IVD than the other haplotypes that mean IVD for Hap5 was 119.0 while the mean IVD for the predominant Hap1 was 111.2 (**Figure 8D**). Hap1 was the haplotype of LOC_Os04g52479 that contains the highest number the rice accessions of RDP1 (**Figure 8E**). According to the 700k SNPs dataset that we used for GWAS, Hap 3 is a synonymous variant that causes base substitution without changing in encoded amino acids. Unfortunately, the available data of the SNPs dataset does not cover all the genetic variants within the NAL1 region, we were unable to provide the variant of Hap5 here. Although the synonymous SNP is a type of non-sense mutation, there are reports of significant impacts of the synonymous SNPs on protein expression and function (Vihinen, 2022). This could be the reason reflecting the higher mean of ML of Hap 3 in our study.

The LD block region for haplotype analysis of the candidate gene *LOC_Os04g48070* (*ROC4*) on chromosome 4 was predicted from 28.523 to 28.614 Mb (91.06 kb) and included 15 SNPs. *LOC_Os04g48070* had a lead SNP 4.28385427 for MNVW (**Supplementary Figure 1**). Nine major haplotypes were detected in the coding region. Hap1 is prevalent in the whole RDP1. We detected that Hap1 is predominant in the TEJ sub-population while Hap2 is prevalent in the TRJ sub-population. Hap1 had a significantly higher MNVW of 27.3 than Hap3 which had a mean MNVW of 24.9. The haplotype analysis of the candidate gene *LOC_Os08g24400* (MED14_1) was conducted using the LD block region estimated to be from 14.686 to 14.784 Mb (97.97 kb) on chromosome 8 and included 13 SNPs. The lead SNP 8.14731173, located in the candidate gene *LOC_Os08g24400* (MED14_1) for MJVW in the RDP1 population. Using the SNPs within the region of *LOC_Os08g24400*, nine major haplotypes were identified (**Supplementary Figure 2**). Hap1 is predominantly in the whole population and TRJ while Hap2 and Hap3 are prevalently detected in IND and TEJ sub-population, respectively. Hap1 had a significantly higher mean MJVW than Hap 2 and Hap3 which is consequent with the high MJVW phenotype detected in TRJ (**Figure 3A**). Further, the candidate gene *LOC_Os02g17390* (*AIM1*) for the VLA trait formed a haplotype block predicted to be from 9.966 to 10.08 Mb (99.32 kb) on chromosome 2 with 24 SNPs which consisted of eleven major alleles. Variation in these haplotypes led to significant phenotypic variation in VLA. Hap1 is prevalent in the whole RDP1 population. TEJ and TRJ are the most abundant sub-population with Hap 1 haplotype. In the whole population, Hap11 had the highest mean VLA of 10.1 which is significantly higher than the mean VLA of the RDP1 population (6.2). Therefore, we defined Hap11 as the favorable haplotype of the vein density trait (**Supplementary Figure 3**).

## Discussion

In this study, three different statistical models, ranging from single to multiple locus models, were compared for GWAS of eight leaf anatomical and vein traits which were different in heritability in 329 accessions of the RDP1. The phenotypic variation among the RDP1 sub-population was relatively high with the coefficients of variant ranging from 9.70% to 51.53% and the heritability ranging from 0.74 to 0.99. Recently, several statistical models for GWAS are available to identify associations of genotypes with numerous phenotypes. Generally, the power of SNP identification power is determined by the population size and structure as well as the heritability influencing the genetic architecture of the trait (Yu et al., 2006; Kaler et al., 2020; Uffelmann et al., 2021). The population structure and a kinship matrix as covariates are incorporated in MLM model that the computation of MLM is intensive. FarmCPU performs marker tests with associated markers as covariates and adopts REML optimization to replace the criterion that the variance explained by kinship is near zero. BLINK is the improved version of the FarmCPU model that Baysian Information criteria (BIC) and linkage disequilibrium approaches are used. In our study, several SNPs were identified by the three models, which implied that all these leaf traits were complex and controlled by an enormous number of genes. Q-Q plots can be used in determining if the models efficiently control false positives and false negatives (Stich et al., 2008; Stich and Melchinger, 2009; Riedelsheimer et al., 2012; Kristensen et al., 2018; Kaler et al., 2020). According to the Q-Q plots of FarmCPU and BLINK, we observed a straight line close to the identity line with a sharp deviated tail, which indicated that these multi-locus models controlled both false positives and false negatives better than MLM, a single locus model, in almost all the traits studied here. However, for ML, IVD, MNVT, and VLA traits, most of the significant markers from the FarmCPU and BLINK were present close to the identity line indicating the increased false negatives which could be generated due to the overfitting of these complex models. For the ML, IVD and MNVT, the FarmCPU and BLINK also exhibited strongly inflated *P* values which implied the contribution of the population structure and the cryptic relationships among individuals of RDP1 in these leaf and vein traits. From our results, Q-Q plots reflected the statistical power of the MLM model in identifying significant SNPs associated with almost all the leaf anatomical and veins traits of the RDP1 sub-population. MLM-based GWAS is an efficient tool that has been successfully used to analyze genetic variation in multiple leaf traits in rice (Hoang et al., 2019; Du et al., 2022).

Understanding leaf development in rice is important for rice yield improvement, as a good leaf shape can optimize rice plant canopy and capture more light thus increasing the photosynthetic efficiency resulting in boosting grain yield. The study of the natural variation of rice leaf thickness revealed a significant positive correlation between leaf thickness and leaf width (Liu et al., 2014). Similarly, the study of the natural variation of rice leaf anatomy showed that thick rice leaves were comprised of wide-diameter veins, while thin leaves were supported by narrow veins (Chatterjee et al., 2016). We found that the number of mesophyll cell layers (ML) in between 2 adjacent minor veins across the leaf was positively related to LT and MNVT. A recent study revealed that the reduction in the number of cell layers observed in bundle sheath cells of leaf veins would account for the thin leaf trait in rice (Chen et al., 2022). The bulked segregants analysis with whole-genome resequencing (BSA-seq) was used in the study to explore insight into the genetic mechanism and identify the quantitative trait loci (QTL) underlying variation of rice leaf thickness. Their results showed that flag leaf thickness is associated with the *qFTL9* in chromosome 9 between 19.10 and 20.03 Mb. Our finding for the MNVT trait that the MLM model reported the significant SNP on chromosome 9 which is located exactly at the candidate region of the *qFTL9* is intriguing. Moreover, for MJVT, BLINK and FarmCPU also reported a similar SNP near LOC_Os09g33690, the candidate gene located within the interval of the *qFTL9*. MLM also identified some correlated SNPs with both the vein’s thickness and width traits that were located in the interval of the *qFTL9*. However, according to our threshold of genome-wide significance, these SNPs were not statistically significant. These results support our finding that flag leaf thickness was strongly positively correlated with the thickness of either minor veins or major veins as well as the number of mesophyll cells layer.

From the numerous numbers of significant loci and candidate genes associated with eight leaf anatomical and vein traits, the three non-synonymous SNPs near *NAL1* detected in ML traits are most intriguing, since the phenotype of *NAL1* deletion mutant is narrow, thicker leaves with increased mesophyll cells layer number (Subudhi et al., 2020). The other remarkable phenotype of the *NAL1* mutant is the reduction in the number of minor veins and the interveinal distance which is consistent with our GWAS results identifying a significant SNP located near *NAL1* associated with the IVD trait. Additionally, a single nucleotide mutation of *NAL1* accounted for variation in the distance between small vascular bundle, flag leaf width, and thickness in rice (Taguchi et al., 2015). Moreover, *BZR4*, the candidate gene for MNVW identified by both BLINK and FarmCPU is upregulated in the *NAL1* deletion mutant which might be responsible for the reduction in the number of minor veins and triggering alteration in leaf width (Subudhi et al., 2020). *NAL1* plays a crucial role in the cell cycle and cell division affecting vein patterning and leaf width since the early stage of leaf primordium initiation and involves in rice yield traits including chlorophyll content, photosynthetic rate, panicle length, and the number of spikelets per panicle (Takai et al., 2010; Fujita et al., 2013; Zhang et al., 2014; Jiang et al., 2015; Lin et al., 2019). In the present study, several significant loci located near the known genes which play important roles in both leaf and panicle development were detected. Among these, the gene *GRAIN NUMBER, PLANT HEIGHT, AND HEADING DATE 7.1* (*GHD7.1*) which regulates flag leaf size and photosynthetic capacity thus improving yield potential in rice (Tang et al., 2018) were significantly identified as a candidate gene for ML trait. Taken all these together, our findings intensify the positive relationship between leaf morphology and yield thus shedding some light on rice molecular breeding which aims to improve yield potential *via* targeting leaf traits improvement. The haplotype analysis results from our study revealed that the haplotypes identified within the LD blocks regulated a diverse range of phenotypic variations in leaf anatomical and vein traits. Therefore, haplotype-based markers can provide more options to modify the desired leaf traits in rice. The incorporation of multiple favorable haplotypes in rice breeding programs can be an effective strategy that will assist in the selection of desirable leaf traits. However, we will need to perform functional validation of the identified candidate genes. Recent advances in molecular technology, such as CRISPR-Cas-based technology are a powerful tool in high-throughput gene editing and provide a rapid method to functionally validate genes and alleles for marker-assisted selection (MAS)-based rice improvement in the future.

## Conclusion

In the present work, we performed GWAS of rice flag leaf traits by using three different statistical models ranging from single to multiple loci including, FarmCPU, BLINK, and MLM. FarmCPU and BLINK performed slightly better than MLM in terms of false-positive controlling. However, MLM was still a powerful model to identify associations of genotypes with flag leaf anatomical and veins traits of the RDP1 sub-population. Here, the MLM-based GWAS identified several significant loci which were associated with the known genes in rice leaf development. Intriguingly, significant SNPs were detected in the interval of *NAL1*, *GHD7.1*, *SLL1*, and some other genes that regulate leaf shape and yield traits. Our findings indicate that flag leaf traits could be improved *via* molecular breeding and can be one of the targets in high-yield rice development.

## Data availability statement

The datasets presented in this study can be found in online repositories. The names of the repository/repositories and accession number(s) can be found in the article/**Supplementary Material**.

## Author contributions

SN designed and performed the experiments in leaf anatomical and vein traits phenotyping. SN also analyzed corresponding data and write the manuscript. B-OT helped grow the rice population throughout the developmental stages and collect leaf samples. YP conducted the association analysis and analyzed GWAS results. PV conceived the project, helped in GWAS data analysis, and provided advice as well as experimental materials. PV also advised on the manuscript concept and prepared the manuscript.
