# Supplementary material for: Multi-model genome-wide association studies of leaf anatomical traits and vein architecture in rice
Source: Front Plant Sci. 2023 Apr 12;14:1107718. doi: 10.3389/fpls.2023.1107718 (PMC10130391; doi:10.3389/fpls.2023.1107718)
Supplement: Supplementary file 1 [file DataSheet_1.docx]

Supplementary Material

Multi-Model Genome-wide Association Studies of Leaf Anatomical Traits and Vein Architecture in Rice

Supatthra Narawatthana^*^, Yotwarit Phansenee , Bang-On Thammasamisorn, Phanchita Vejchasarn

*** Correspondence:** Supatthra Narawatthana: supatthra.n@rice.mail.go.th

# Supplementary Figures and Tables

## Supplementary Figures


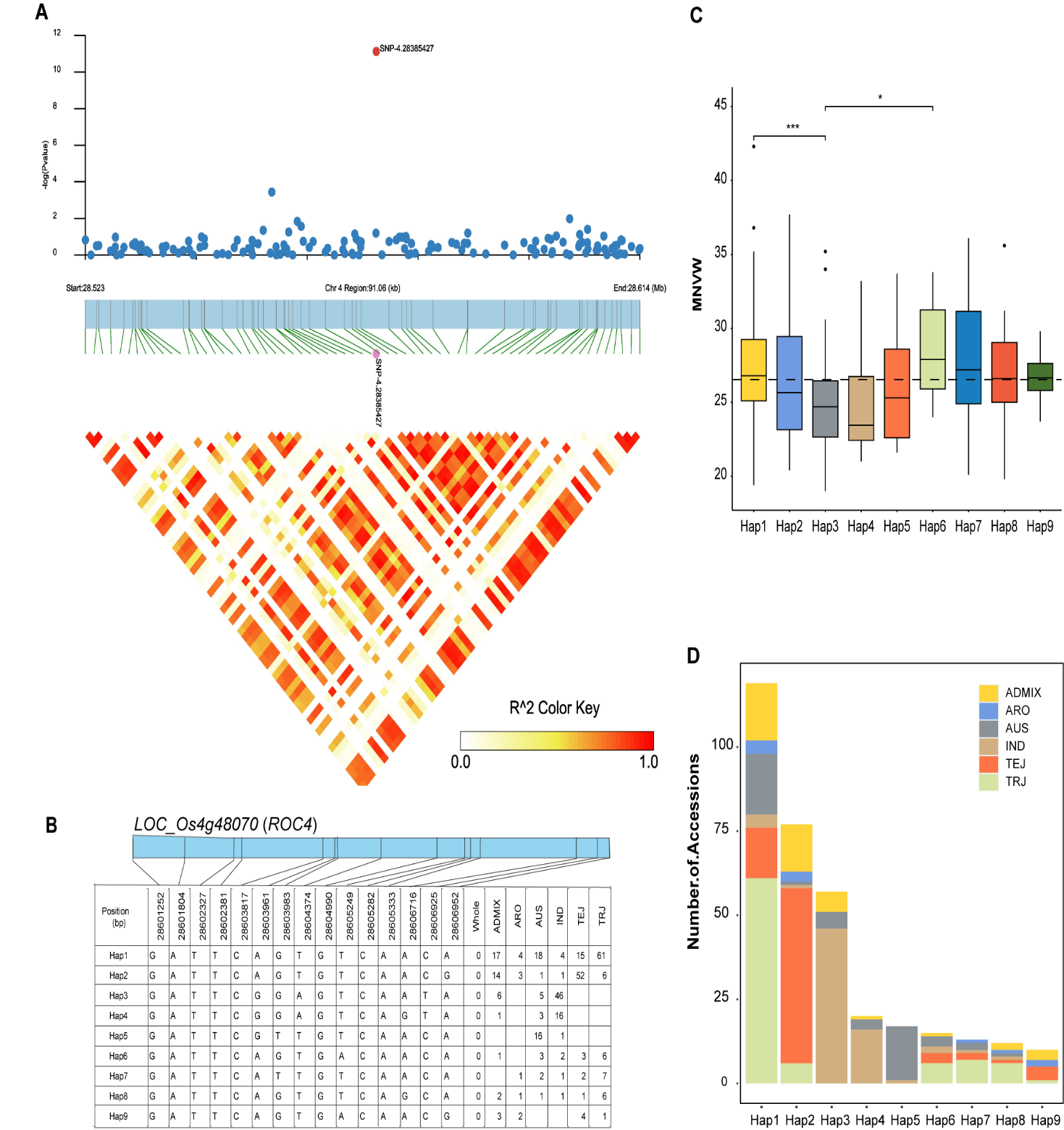
**Supplementary Figure 1.** Haplotype analysis of *LOC_Os4g48070* (*ROC4*). (A) Local Manhattan plot (top) and LD heatmap (bottom) of a locus associated with minor vein width (MNVW) in RDP1 sub-population. The red dot indicates the lead SNP 4.28385427. (B) Haplotypes of *LOC_Os4g48070*. (C) The distribution of MNVW in RDP1 sub-population for the nine haplotypes of *LOC_Os4g48070*. Asterisk indicates significant differences among haplotypes according to Kruskal-Wallis test and the pairwise Wilcoxon test (*P* < 0.05). (D) Frequency of the five haplotypes of *LOC_Os4g48070* in RDP1 sub-population. The black dot indicates the outlier data.


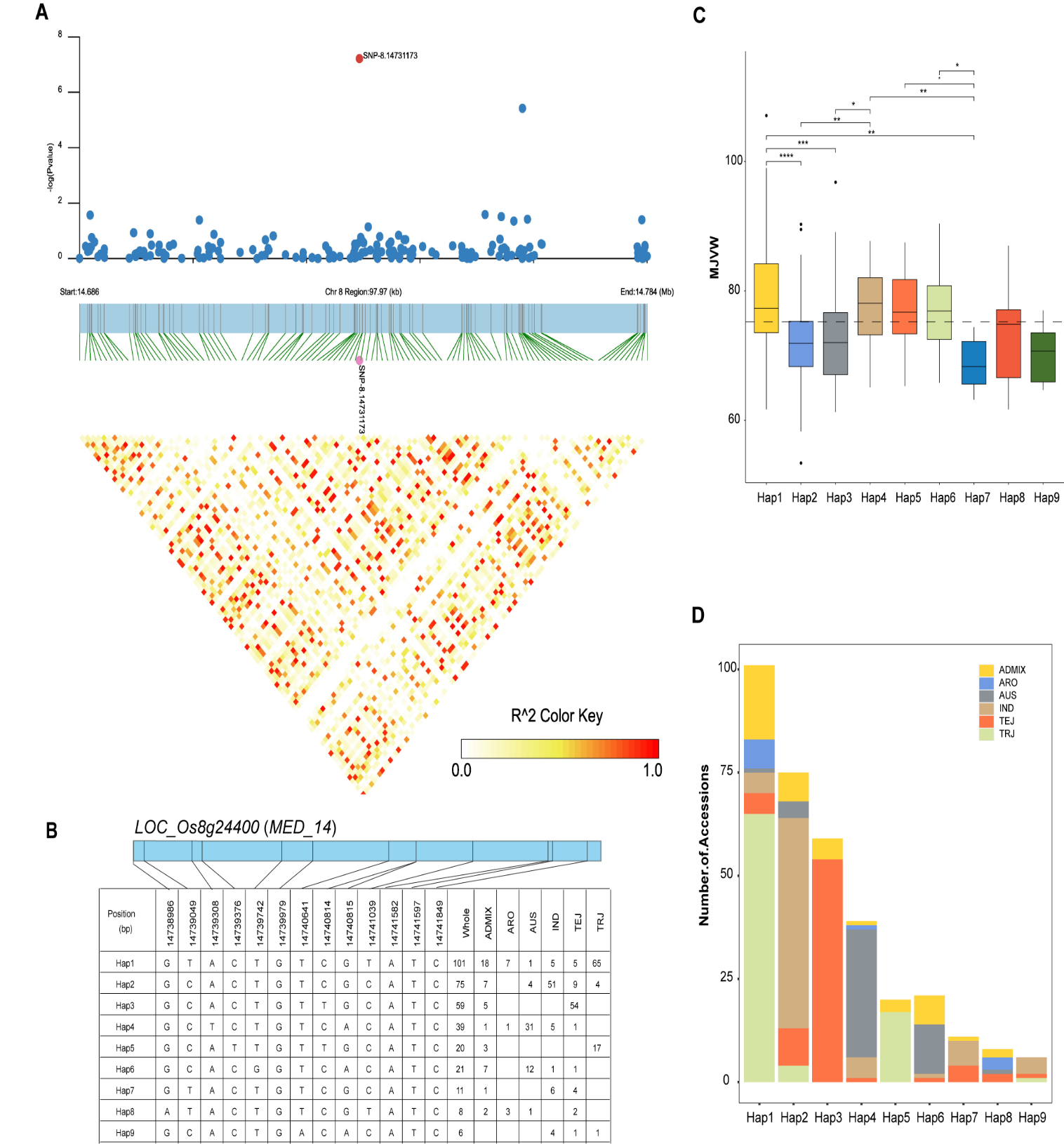
**Supplementary Figure 2.** Haplotype analysis of *LOC_Os8g24400* (*MED_14*). (A) Local Manhattan plot (top) and LD heatmap (bottom) of a locus associated with major vein width (MJVW) in RDP1 sub-population. The red dot indicates the lead SNP 8.14731173. (B) Haplotypes of *LOC_Os8g24400*. (C) The distribution of MNVW in RDP1 sub-population for the nine haplotypes of *LOC_Os8g24400*. Asterisk indicates significant differences among haplotypes according to Kruskal-Wallis test and the pairwise Wilcoxon test (*P* < 0.05). (D) Frequency of the five haplotypes of *LOC_ Os8g24400* in RDP1 sub-population. The black dot indicates the outlier data.

**Supplementary Figure 3.** Haplotype analysis of *LOC_Os2g17390* (*AIM1*). (A) Local Manhattan plot (top) and LD heatmap (bottom) of a locus associated with vein density or vein length per leaf area (VLA) in RDP1 sub-population. The red dot indicates the lead SNP 2.10015824. (B) Haplotypes of *LOC_Os2g17390*. (C) The distribution of MNVW in RDP1 sub-population for the 11 haplotypes of *LOC_Os2g17390*. Asterisk indicates significant differences among haplotypes according to Kruskal-Wallis test and the pairwise Wilcoxon test (*P* < 0.05). (D) Frequency of the five haplotypes of *LOC_Os2g17390*in RDP1 sub-population. The black dot indicates the outlier data
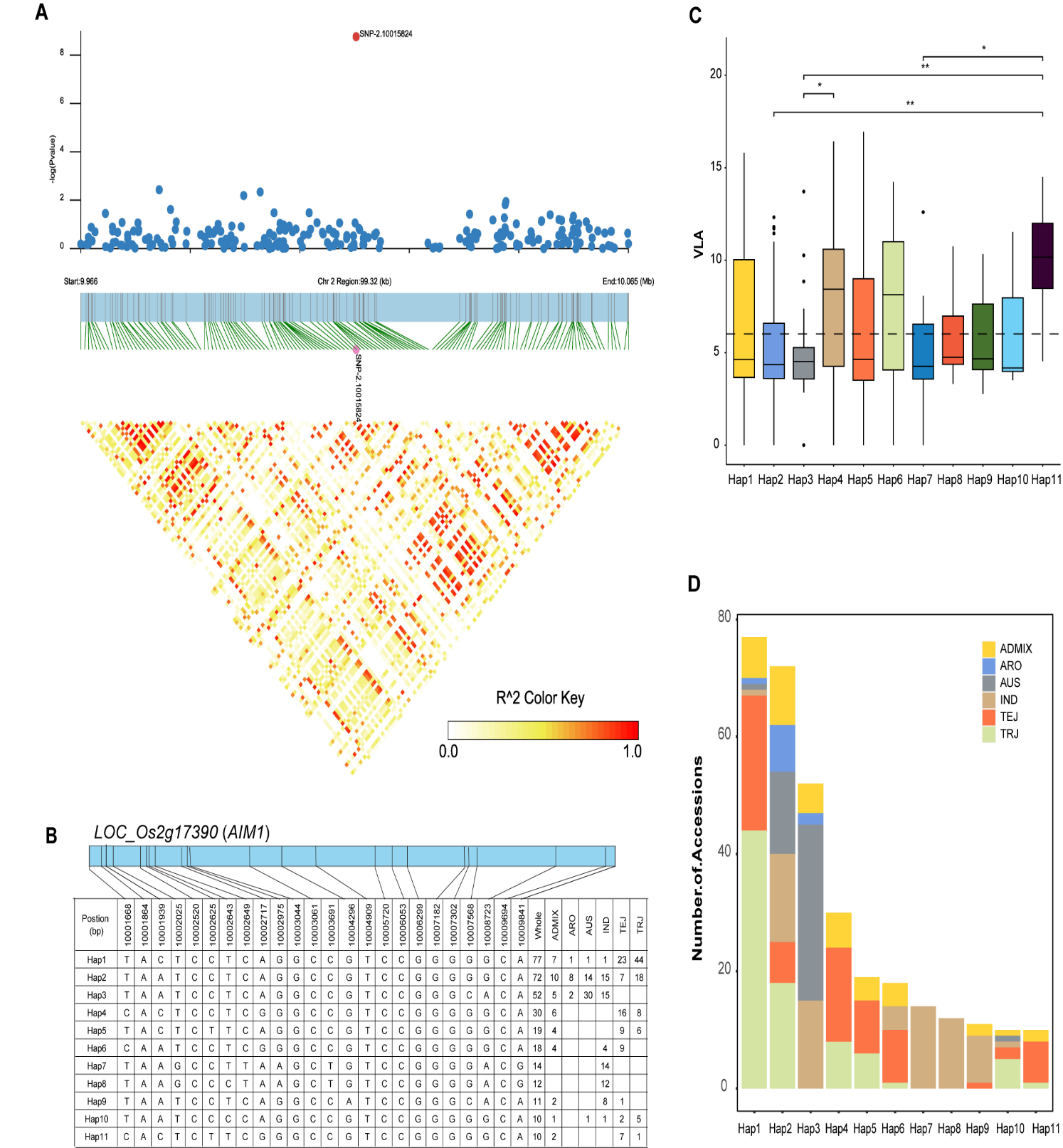
.

## Supplementary Table

**Supplementary Table S1 General information of 329 rice accessions of RDP1.**

| IRGC ID^¶^ | Name | Orignal providing country | Subpopulation group (fastStructure)^¶¶^ |
| --- | --- | --- | --- |
| 117264 | Azucena | Philippines | tropical-japonica |
| 117265 | Dom Sufid | Iran | aromatic* |
| 117266 | Dular | India | aus |
| 117268 | IR64-21 | Philippines | indica |
| 117269 | Li-Jiang-Xin-Tuan-Hei-Gu | China | temperate-japonica |
| 117270 | M-202 | United States | temperate-japonica* |
| 117271 | Minghui 63 | China | indica |
| 117273 | N 22 | India | aus* |
| 117274 | Nipponbare | Japan | temperate-japonica |
| 117276 | Sadu Cho | Republic of Korea | indica |
| 117277 | Shan-Huang-Zhan-2 | China | indica* |
| 117279 | Tainung 67 | Taiwan | temperate-japonica |
| 117280 | Zhenshan 97B | China | indica |
| 117282 | Cypress | United States | tropical-japonica |
| 117586 | Tchampa ^##^ | Iran | temperate-japonica* |
| 117599 | 27 | Dominican Republic | tropical-japonica |
| 117600 | Aijiaonante | China | indica |
| 117601 | NSF-TV 4 | Unknown | aus |
| 117602 | ARC 6578 | India | aus |
| 117603 | ARC 7229 | India | aus |
| 117604 | ASD 1 | India | temperate-japonica |
| 117606 | C57-5043 | United States | tropical-japonica |
| 117607 | Carolina Gold 12033 | United States | tropical-japonica |
| 117608 | Carolina Gold Sel | United States | tropical-japonica |
| 117609 | CS-M3 | United States | temperate-japonica |
| 117610 | DZ78 | Bangladesh | aus* |
| 117611 | Gerdeh | Iran | admixed-japonica |
| 117612 | Geumobyeo | Republic of Korea | temperate-japonica |
| 117613 | Habiganj Boro 6 | Bangladesh | admixed |
| 117614 | Hon Chim | Hong Kong | indica* |
| 117615 | IAC 25 | Brazil | tropical-japonica |
| 117616 | Italica Carolina | Poland | temperate-japonica |
| 117617 | Kasalath | India | aus |
| 117618 | Keriting Tingii | Indonesia | admixed-japonica |
| 117619 | Leung Pratew | Thailand | indica |
| 117620 | Mehr | Iran | aus |
| 117621 | Moroberekan | Guinea | tropical-japonica |
| 117622 | RTS14 | Vietnam | indica |
| 117623 | BR24 | Bangladesh | indica |
| 117624 | Kiuki No. 46 | Japan | temperate-japonica |
| 117625 | SML 242 | Suriname | indica |
| 117626 | WC 4419 | Honduras | tropical-japonica |
| 117628 | NSF-TV 260 | Unknown | aromatic |
| 117629 | 318 | Turkey | tropical-japonica |
| 117630 | 519 | Uruguay | indica |
| 117631 | 56-122-23 | Thailand | temperate-japonica |
| 117632 | 583 | Ecuador | tropical-japonica |
| 117633 | 68-2 | France | temperate-japonica |
| 117634 | 9524 | India | aus |
| 117635 | Agusita | Hungary | temperate-japonica |
| 117636 | Ai-Chiao-Hong | China | indica |
| 117638 | Amposta | Puerto Rico | temperate-japonica |
| 117639 | Arabi | Egypt | admixed-japonica |
| 117640 | ARC 10086 | India | tropical-japonica |
| 117641 | NSF-TV 5 | Unknown | aromatic |
| 117642 | ARC 10376 | India | aus |
| 117643 | Asse Y Pung | Philippines | tropical-japonica |
| 117644 | Aswina 330 | Bangladesh | aus* |
| 117645 | Azerbaidjanica | Azerbaijan | temperate-japonica |
| 117647 | Baber | India | temperate-japonica |
| 117648 | Baghlani Nangarhar | Afghanistan | temperate-japonica |
| 117649 | Bahia | Spain | temperate-japonica |
| 117651 | Baldo | Italy | admixed-japonica |
| 117652 | Basmati | Pakistan | aromatic* |
| 117653 | Basmati 217 | India | aromatic* |
| 117654 | Bellardone | France | temperate-japonica |
| 117655 | Benllok | Peru | temperate-japonica |
| 117656 | Berenj | Afghanistan | admixed |
| 117658 | Bico Branco | Brazil | aromatic |
| 117659 | Binulawan | Philippines | indica |
| 117660 | Biser 1 | Bulgaria | temperate-japonica |
| 117661 | BJ 1 | India | aus |
| 117662 | Black Gora | India | aus |
| 117663 | Blue Rose | United States | admixed-japonica |
| 117664 | Boa Vista | El Salvador | tropical-japonica |
| 117665 | Bombilla | Spain | temperate-japonica |
| 117666 | Bombon | Spain | temperate-japonica |
| 117667 | Breviaristata | Portugal | admixed-japonica |
| 117668 | British Honduras Creole | Belize | tropical-japonica |
| 117669 | Bul Zo | Republic of Korea | temperate-japonica |
| 117670 | Bulgare | France | temperate-japonica |
| 117671 | Byakkoku Y 5006 Seln | Australia | indica |
| 117672 | C1-6-5-3 | Mexico | admixed |
| 117673 | CA 902/B/2/1 | Chad | aus |
| 117674 | Caawa/Fortuna 6-103-15 | Taiwan | tropical-japonica |
| 117675 | Canella De Ferro | Brazil | tropical-japonica |
| 117676 | Carolina Gold 12034 | United States | tropical-japonica |
| 117677 | Caucasica | Former Soviet Union | temperate-japonica |
| 117678 | Cenit | Argentina | tropical-japonica |
| 117679 | NSF-TV 27 | Unknown | tropical-japonica |
| 117681 | Chang Ch'Sang Hsu Tao | China | indica |
| 117682 | Chau | Vietnam | indica |
| 117683 | Chibica | Mozambique | temperate-japonica |
| 117685 | China 1039 | China | indica |
| 117686 | Chinese | China | temperate-japonica |
| 117687 | Chodongji | Republic of Korea | temperate-japonica |
| 117688 | Chuan 4 | Taiwan | aus |
| 117689 | CI 11011 | United States | tropical-japonica |
| 117690 | CI 11026 | United States | admixed |
| 117691 | CO18 | India | indica |
| 117692 | Coarse | Pakistan | aus |
| 117693 | Cocodrie | United States | tropical-japonica |
| 117694 | Coppocina | Bulgaria | tropical-japonica |
| 117695 | Creole | Belize | tropical-japonica |
| 117696 | Criollo La Fria | Venezuela | indica |
| 117697 | CTG 1516 | Bangladesh | aus |
| 117698 | Cuba 65 | Cuba | tropical-japonica |
| 117699 | Cybonnet | United States | tropical-japonica |
| 117700 | NSF-TV 39 | Unknown | aus |
| 117701 | Dam | Thailand | admixed-japonica |
| 117702 | Darmali | Nepal | admixed |
| 117703 | Dawebyan | Myanmar | indica |
| 117704 | DD 62 | Bangladesh | aus |
| 117705 | Dee Geo Woo Gen | Taiwan | indica |
| 117706 | Della | United States | tropical-japonica |
| 117707 | Delrex | United States | tropical-japonica |
| 117708 | Deokjeokjodo | Republic of Korea | temperate-japonica |
| 117709 | Desvauxii | Former Soviet Union | temperate-japonica |
| 117710 | Dhala Shaitta | Bangladesh | aus |
| 117711 | DJ 123 | Bangladesh | aus |
| 117712 | DJ 24 | Bangladesh | aus |
| 117713 | Djimoron | Guinea | indica |
| 117714 | DK 12 | Bangladesh | aus |
| 117715 | DM 43 | Bangladesh | aus |
| 117716 | DM 56 | Bangladesh | aus |
| 117717 | DM 59 | Bangladesh | aus |
| 117718 | DNJ 140 | Bangladesh | aus |
| 117719 | Doble Carolina Rinaldo Barsani | Uruguay | admixed-japonica |
| 117720 | Dom Zard | Iran | aromatic |
| 117721 | Dom Sufid | Iran | aromatic |
| 117722 | Dosel | Spain | temperate-japonica |
| 117723 | Dourado Agulha | Brazil | tropical-japonica |
| 117724 | DV 123 | Bangladesh | aus |
| 117725 | DV85 | Bangladesh | aus |
| 117727 | Early Wataribune | Japan | temperate-japonica |
| 117728 | ECIA76-S89-1 | Cuba | indica |
| 117729 | Edith | United States | tropical-japonica |
| 117731 | EMATA A 16-34 | Myanmar | indica |
| 117732 | Erythroceros Hokkaido | Poland | temperate-japonica |
| 117733 | Estrela | Colombia | admixed-japonica |
| 117735 | Firooz | Iran | aromatic |
| 117736 | Fortuna | United States | tropical-japonica |
| 117737 | Fossa Av | Burkina Faso | tropical-japonica |
| 117739 | NSF-TV 57 | Unknown | indica |
| 117740 | Ghati Kamma Nangarhar | Afghanistan | aus |
| 117741 | Ghorbhai | Bangladesh | aus |
| 117743 | Goria | Bangladesh | aus |
| 117744 | Gotak Gatik | Indonesia | admixed-japonica |
| 117745 | Guan-Yin-Tsan | China | indica* |
| 117746 | Guineandao | Guinea | admixed-japonica |
| 117747 | Halwa Gose Red | Iraq | aus |
| 117748 | Hatsunishiki | Japan | temperate-japonica |
| 117749 | Hiderisirazu | Japan | admixed-japonica |
| 117750 | Honduras | Honduras | tropical-japonica |
| 117751 | Hsia Chioh Keh Tu | Taiwan | indica |
| 117752 | Hu Lo Tao | China | temperate-japonica |
| 117753 | Hunan Early Dwarf No. 3 | China | indica |
| 117754 | I-Geo-Tze | Taiwan | indica* |
| 117755 | Iguape Cateto | Haiti | tropical-japonica |
| 117756 | IITA 135 | Nigeria | tropical-japonica |
| 117757 | IR 36 | Philippines | indica |
| 117758 | IR 8 | Philippines | indica |
| 117759 | IR-44595 | Nepal | indica |
| 117760 | IRAT 13 | Cote D'Ivoire | tropical-japonica |
| 117761 | IRAT 177 | French Guiana | tropical-japonica |
| 117762 | IRAT 44 | Burkina Faso | tropical-japonica |
| 117765 | Jamir | Bangladesh | aus |
| 117766 | Jaya | India | indica |
| 117767 | JC 117 | India | indica |
| 117768 | JC149 | India | indica |
| 117769 | Jhona 349 | India | aus |
| 117770 | JM70 | Mali | indica |
| 117771 | Jouiku 393G | Japan | temperate-japonica |
| 117772 | Kachilon | Bangladesh | aus |
| 117773 | Kalamkati | India | aus |
| 117774 | Kalubala Vee | Sri Lanka | aus |
| 117775 | Kamenoo | Japan | temperate-japonica |
| 117776 | Kaniranga | Indonesia | tropical-japonica |
| 117777 | Karabaschak | Bulgaria | temperate-japonica |
| 117778 | Karkati 87 | Bangladesh | aus |
| 117779 | Kaukau | Mali | aus |
| 117780 | Kaukkyi Ani | Myanmar | admixed-japonica* |
| 117781 | Khao Gaew | Thailand | aus* |
| 117782 | Khao Hawm | Thailand | tropical-japonica |
| 117784 | Kiang-Chou-Chiu | Taiwan | indica |
| 117785 | Kihogo | Tanzania | temperate-japonica |
| 117786 | Kinastano | Philippines | tropical-japonica |
| 117788 | Kon Suito | Mongolia | admixed |
| 117789 | Koshihikari | Japan | temperate-japonica |
| 117791 | KPF-16 | Bangladesh | admixed-indica |
| 117792 | KU115 | Thailand | tropical-japonica |
| 117793 | Kun-Min-Tsieh-Hunan | China | indica* |
| 117794 | L-202 | United States | tropical-japonica |
| 117795 | La 110 | United States | indica* |
| 117796 | LAC 23 | Liberia | tropical-japonica |
| 117797 | Lacrosse | United States | admixed-japonica |
| 117798 | Lady Wright Seln | United States | tropical-japonica |
| 117799 | Lambayeque 1 | Peru | aromatic |
| 117800 | LD 24 | Sri Lanka | indica* |
| 117801 | Leah | United States | tropical-japonica |
| 117802 | Lemont | United States | tropical-japonica |
| 117803 | Leuang Hawn | Thailand | temperate-japonica |
| 117804 | Ligerito | Colombia | tropical-japonica |
| 117805 | Llanero 501 | Venezuela | tropical-japonica |
| 117806 | Lomello | Italy | temperate-japonica |
| 117807 | Luk Takhar | Afghanistan | temperate-japonica |
| 117808 | Lusitano | Portugal | temperate-japonica |
| 117809 | M-202 | United States | admixed-japonica |
| 117810 | M. Blatec | Macedonia | temperate-japonica |
| 117811 | Mansaku | Japan | temperate-japonica |
| 117812 | Maratelli | Italy | temperate-japonica |
| 117814 | Ming Hui | China | indica |
| 117815 | NSF-TV 107 | Unknown | tropical-japonica |
| 117816 | Mojito Colorado | Bolivia | tropical-japonica |
| 117817 | MTU9 | India | indica* |
| 117818 | Mudgo | India | indica |
| 117819 | N12 | India | aromatic |
| 117820 | Niquen | Chile | temperate-japonica* |
| 117822 | Norin 20 | Japan | temperate-japonica |
| 117823 | Nova | United States | admixed-japonica |
| 117824 | NSF-TV 116 | Unknown | tropical-japonica |
| 117825 | Nucleoryza | Austria | temperate-japonica |
| 117826 | O-Luen-Cheung | Taiwan | indica |
| 117827 | Okshitmayin | Myanmar | admixed-japonica |
| 117828 | Oro | Chile | temperate-japonica |
| 117829 | Oryzica Llanos 5 | Colombia | indica |
| 117830 | OS 6 (WC 10296) | Zaire | tropical-japonica |
| 117831 | OS6 | Nigeria | tropical-japonica |
| 117833 | Ostiglia | Italy | admixed-japonica |
| 117834 | P 737 | Pakistan | aus |
| 117835 | Padi Kasalle | Indonesia | tropical-japonica |
| 117836 | Padi Pagalong | Malaysia | tropical-japonica |
| 117837 | Pagaiyahan | Taiwan | indica |
| 117838 | Pai Hok Glutinous | Hong Kong | indica |
| 117839 | Palmyra | United States | tropical-japonica |
| 117840 | Pao-Tou-Hung | China | indica |
| 117841 | Pappaku | Taiwan | indica |
| 117842 | Paraiba Chines Nova | Brazil | indica |
| 117843 | Pate Blanc Mn 1 | Cote D'Ivoire | tropical-japonica |
| 117844 | Patna | Morocco | admixed-japonica |
| 117846 | Pato De Gallinazo | Australia | admixed-japonica |
| 117847 | Paung Malaung | Myanmar | aus |
| 117849 | Peh-Kuh-Tsao-Tu | Taiwan | indica |
| 117850 | Phudugey | Bhutan | aus |
| 117851 | PI 298967-1 | Australia | admixed-japonica |
| 117853 | PR 304 | Puerto Rico | tropical-japonica |
| 117854 | Pratao | Brazil | tropical-japonica |
| 117855 | Priano Guaira | Brazil | tropical-japonica |
| 117856 | PTB 30 | India | aus |
| 117857 | R 101 | Zaire | tropical-japonica |
| 117858 | Radin Ebos 33 | Malaysia | indica |
| 117859 | Rathuwee | Sri Lanka | indica |
| 117860 | Razza 77 | Italy | temperate-japonica |
| 117861 | Rikuto Kemochi | Japan | temperate-japonica |
| 117862 | Rikuto Norin 21 | Japan | admixed-japonica |
| 117863 | Rinaldo Bersani | Italy | temperate-japonica |
| 117864 | Riz Local | Burkina Faso | admixed-indica |
| 117865 | Rojofotsy 738 | Madagascar | admixed-indica |
| 117866 | Romanica | Hungary | temperate-japonica |
| 117867 | RT 1031-69 | Zaire | tropical-japonica* |
| 117868 | RTS4 | Vietnam | indica |
| 117869 | S4542A3-49B-2B12 | United States | tropical-japonica |
| 117871 | Sabharaj | Bangladesh | indica |
| 117872 | Sadri Belyi | Azerbaijan | aromatic |
| 117873 | Sadri Tor Misri | Iran | admixed-indica |
| 117874 | Saku | Mongolia | tropical-japonica |
| 117875 | Sanbyang-Daeme | Republic of Korea | admixed-japonica |
| 117876 | Santhi Sufaid | Pakistan | aus |
| 117877 | Saraya | Fiji | aus |
| 117878 | Sathi | Pakistan | aus |
| 117879 | Saturn | United States | tropical-japonica |
| 117880 | Seratoes Hari | Indonesia | indica |
| 117881 | Shai-Kuh | China | indica |
| 117883 | Shim Balte | Iraq | aus |
| 117884 | Shinriki | Japan | temperate-japonica |
| 117885 | Shirkati | Afghanistan | aus |
| 117886 | Shirogane | Japan | temperate-japonica |
| 117887 | Shoemed | United States | temperate-japonica |
| 117888 | Short Grain | Thailand | indica |
| 117889 | Sigadis | Indonesia | indica |
| 117890 | Sinampaga Selection | Philippines | tropical-japonica |
| 117891 | Sintane Diofor | Burkina Faso | indica |
| 117892 | Sitpwa | Myanmar | temperate-japonica |
| 117893 | SL 22-613 | Sierra Leone | indica |
| 117894 | SLO 17 | India | indica |
| 117895 | Sri Malaysia Dua | Malaysia | temperate-japonica |
| 117896 | Sufaid | Pakistan | aus |
| 117897 | Sultani | Egypt | tropical-japonica |
| 117898 | Sundensis | Kazakhstan | indica |
| 117899 | Surjamkuhi | India | aus |
| 117900 | Suweon 362 | Republic of Korea | temperate-japonica |
| 117901 | Sze Guen Zim | China | indica |
| 117902 | T 1 | India | aus |
| 117903 | T26 | India | aus |
| 117904 | Ta Hung Ku | China | temperate-japonica |
| 117905 | Ta Mao Tsao | China | temperate-japonica |
| 117906 | Taducan | Philippines | indica |
| 117907 | Taichung Native 1 | Taiwan | indica |
| 117908 | Tainan Iku 487 | Taiwan | temperate-japonica |
| 117909 | Tainan-Iku No. 512 | Taiwan | temperate-japonica |
| 117910 | Taipei 309 | Taiwan | temperate-japonica |
| 117911 | Tchibanga | Gabon | indica |
| 117912 | TeQing | China | indica |
| 117914 | Tia Bura | Indonesia | tropical-japonica |
| 117915 | TKM6 | India | indica |
| 117916 | Tog 7178 | Senegal | admixed-indica |
| 117917 | Tokyo Shino Mochi | Japan | admixed-japonica |
| 117918 | Tondok | Indonesia | tropical-japonica |
| 117919 | Toploea 70/76 | Romania | temperate-japonica |
| 117920 | Tox 782-20-1 | Nigeria | tropical-japonica |
| 117921 | Trembese | Indonesia | tropical-japonica |
| 117923 | Tropical Rice | Ecuador | temperate-japonica |
| 117924 | Tsipala 421 | Madagascar | admixed-indica |
| 117925 | Uzbekskij 2 | Uzbekistan | temperate-japonica |
| 117926 | Vary Vato 462 | Madagascar | admixed-indica* |
| 117927 | Varyla | Madagascar | tropical-japonica |
| 117928 | Vavilovi | Kazakhstan | temperate-japonica |
| 117929 | Vialone | Italy | admixed-japonica |
| 117931 | WAB 501-11-5-1 | Cote D'Ivoire | tropical-japonica |
| 117932 | WAB 502-13-4-1 | Cote D'Ivoire | tropical-japonica |
| 117935 | WC 3397 | Jamaica | tropical-japonica |
| 117936 | WC 4443 | Bolivia | tropical-japonica |
| 117937 | WC 521 | China | admixed-japonica |
| 117938 | WIR 3764 | Uzbekistan | temperate-japonica |
| 117940 | Yodanya | Myanmar | indica |
| 117941 | YRL-1 | Australia | admixed-japonica |
| 117942 | Zerawchanica Karatalski | Poland | temperate-japonica |
| 117943 | ZHE 733 | China | indica |
| 117944 | Zhenshan 2 | China | indica |
| 121652 | H256-76-1-1-1 | Argentina | tropical-japonica |
| 121653 | Khao Pahk Maw | Thailand | aus |
| 124370 | 325 | Taiwan | tropical-japonica |
| 124371 | Blue Rose Supreme | United States | admixed-japonica |
| 124372 | Manzano | Zaire | tropical-japonica |
| 124373 | Nortai | United States | admixed-japonica |
| 124374 | Romeo | Italy | temperate-japonica |
| 124375 | Sinaguing | Philippines | tropical-japonica |
| 124376 | Sung Liao 2 | China | temperate-japonica |
| 125595 | Shuang-Chiang | Taiwan | indica* |
| 125596 | C101A51 | Philippines | indica |
| 125597 | Jasmine85 | Philippines | indica |
| 125598 | Jing 185-7 | China | indica |
| 125599 | Wanica | Suriname | tropical-japonica |
| 126353 | 923 | Madagascar | admixed |
| 126356 | Beonjo | Republic of Korea | temperate-japonica |
| 126365 | Haginomae Mochi | Japan | temperate-japonica |
| 126368 | Kaw Luyoeng | Thailand | temperate-japonica* |
| 126377 | Tam Cau 9A | Vietnam | indica |
| 126380 | Agostano | Italy | temperate-japonica |
| 126382 | Early | United States | admixed-japonica |
| 126383 | Guatemala 1021 | Guatemala | tropical-japonica |
| 126384 | Heukgyeong | Republic of Korea | temperate-japonica |
| 126385 | Jefferson | United States | tropical-japonica |
| 126386 | Katy | United States | tropical-japonica |
| 126387 | Kaybonnet | United States | tropical-japonica |
| 126388 | LaGrue | United States | tropical-japonica |
| 126389 | Pecos | United States | admixed-japonica |
| 126390 | Rondo (4484-1693) | China | indica |
| 126391 | Rosemont | United States | tropical-japonica |
| 126392 | RT0034 | United States | indica |
| 126393 | Saber | United States | tropical-japonica |
| 126394 | Shufeng 121-1655 | China | indica* |
| 126395 | Stegaru 65 | Romania | temperate-japonica |
| 126396 | Upland | Ponape Island, Micronesia | tropical-japonica |
| 126399 | NPE 835 | Pakistan | temperate-japonica |
| 126400 | NSF-TV 34 | Unknown | indica |
| 126401 | Pankhari 203 | India | aromatic |
| 126402 | Bengal | United States | admixed-japonica |
| 126403 | Gyehwa 3 | Republic of Korea | temperate-japonica |
| 126404 | Panda | Italy | admixed-japonica |
| 126405 | Sml Kapuri | Suriname | temperate-japonica |

¶ IRGC ID.: International Rice Germplasm Collection identification number.

¶¶ Subpopulation identified by fastStructure analysis based on 700,000 SNPs (McCouch et al., 2016).

* Subpopulation identified by STRUCTURE analysis based on 36 SSRs (Ali et al., 2011).

##an accession from the Rice Diversity Panel 2 (RDP2) collection (McCouch et al. ,2016).
